# Design, Analysis, and Prototyping of a Multifunctional Digital Twin-Enabled Aerospace Drilling End-Effector Deployable by a Collaborative Robot

**DOI:** 10.3390/s25247504

**Published:** 2025-12-10

**Authors:** Mahdi Kazemiesfahani, Erfan Dilfanian, Bruno Monsarrat, Seyedhossein Hajzargarbashi

**Affiliations:** 1 Aerospace Manufacturing Technologies Centre, National Research Council Canada, Montreal, QC H3T 1J4, Canada; mahdi.kazemiesfahani@cnrc-nrc.gc.ca (M.K.); erfan.dilfanian@nrc-cnrc.gc.ca (E.D.); bruno.monsarrat@cnrc-nrc.gc.ca (B.M.); 2 Department of Mechanical Engineering, University of Manitoba, Winnipeg, MB R3T 5V6, Canada; 3 Department of Mechanical, Industrial and Aerospace Engineering, Concordia University, Montreal, QC H3G 1M8, Canada

**Keywords:** collaborative robot (cobot), one-up assembly, automated drilling, cyber–physical system (CPS), digital twin, ROS, End-of-Arm Tool (EoAT), Industry 4.0, ACME

## Abstract

Drilling in aerospace one-up assembly demands high positional accuracy, strong clamping forces, and precise angular compensation to ensure quality in multi-layered stacks. Existing robotic solutions achieve these requirements but are costly, bulky, and unsuitable for flexible or collaborative environments. This work introduces the Advanced Collaborative Multifunctional End-Effector (ACME), a lightweight robotic drilling end-effector designed for integration with collaborative robots (cobots). ACME incorporates vacuum-assisted clamping capable of generating high forces, a passive self-normalization mechanism for angular alignment on double-curvature surfaces, and a compact 5-DoF positioning system for precise positioning and orientation. The system’s kinematics and dynamics were modeled and experimentally verified through frequency response function (FRF) testing, enabling precise behavior prediction. The tool is integrated within a cyber–physical system (CPS) featuring an interactive digital twin that, unlike passive monitoring systems, allows operators to configure workpieces, select drilling locations directly from rendered CAD, and supervise execution without programming expertise. Experiments demonstrated average positional errors of 0.19 mm and normality deviations of 0.29°, both within aerospace standards. The results confirm that ACME effectively extends cobot capabilities for aerospace-grade drilling while improving flexibility, safety, and operator accessibility.

## 1. Introduction

Drilling is one of the most critical processes in aerospace manufacturing, directly affecting productivity, quality, and safety. Modern aircraft structures require hundreds of thousands of precision-drilled holes in various combinations of multi-layered stacks, including aluminum, titanium, and carbon fiber-reinforced polymer (CFRP). Each hole must be executed with high positional accuracy, strict angular normality, and sufficient clamping force to prevent interlayer burr formation or delamination. Prior research has emphasized that adequate clamping force is a decisive factor in ensuring aerospace drilling quality. Studies [1,2,3,4] demonstrated that controlling clamping loads between 500 and 1500 N minimizes interlayer gaps, reduces burr formation, and prevents surface/interface damage, particularly in CFRP/Ti stacks where higher forces are required. Pardo [5] further highlighted that tuning drilling forces, parameters, and cooling strategies improves borehole integrity and reduces tool wear in multi-material assemblies. This shows the significance of providing and maintaining high forces for drilling, which requires high-payload automated systems. Maintaining drilling normality is equally critical, as aerospace standards typically restrict angular deviations to within ±0.5° [6]. To address this, several solutions have been proposed in the literature. Jie and Shu-Hui [7] as well as Shi et al. [8] introduced mechanical and laser-based correction methods, employing eccentric disks and real-time orientation adjustment systems to ensure alignment during complex drilling tasks. Chen et al. [9] further demonstrated the effectiveness of laser-sensor-based orientation systems in compensating for variations on double-curvature surfaces, enabling consistent normality across non-planar geometries. Yuan et al. [10] developed a machine learning algorithm that models the positional error and compensates for that without live sensor data, thereby reducing system complexity while enhancing accuracy. In addition, an “auto-normalization” method using laser sensors [11] has been developed to determine the surface normal and dynamically adjust drilling orientation, providing a robust pathway for precision drilling in challenging aerospace assemblies. While active normalization systems have shown promising results, they often introduce added complexity, cost, and reliability concerns. These approaches typically rely on closed-loop feedback from sensors and motorized orientation units that must continuously adjust the drilling angle during operation. Such mechanisms can become prone to backlash or locking when subjected to high clamping forces, particularly in multi-material stacks requiring loads in the kilonewton range. On double-curvature surfaces, the real-time computation and motor synchronization required to maintain tool normality can also lead to control instability, slow response times, and potential surface damage if the feedback loop lags.

The introduction of a passive normalization mechanism would represent a major advancement in automated aerospace drilling. If such a system were to be realized, it could inherently align the drilling axis with the local surface normal as the clamping force builds up, without relying on additional actuators or continuous feedback. This would drastically simplify the system control architecture and mechanisms, reduce power consumption and maintenance requirements, and enhance reliability under high-load conditions. Moreover, a passive approach would naturally adapt to variations in fuselage geometry, making it particularly advantageous for double-curvature surfaces where maintaining normality is most challenging. The absence of such mechanisms in existing literature highlights a critical gap in developing an inherently simple and stable alternative.

**Figure 1:**
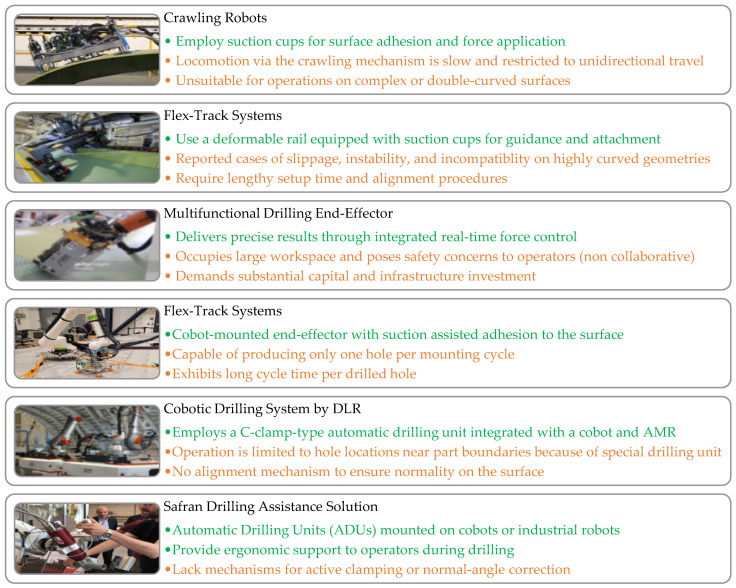
Strengths (in green) and Drawbacks (in orange) of State-of-the-Art Automated Drilling Technologies.

Traditional manual drilling methods remain common but are increasingly impractical due to their labor intensity, ergonomic risks, variability in quality, and inability to scale to production demands. Industrial robotic drilling platforms address these issues by providing high precision, repeatability, and integration with automated workflows. The strengths and drawbacks of robotic drilling solutions like Marguet et al. [12], Cirillo et al. [13], MTORRES crawling robot [14], MTM Robotics’ Mini FlexTrack [15], and integrated cobot–Automatic Drilling Unit (ADU) by SAFRAN [16] are illustrated in Figure 1. Crawling robots, collaborative units, and flex-track structures offer clamping forces, positioning, and normality adaptation for various geometries. Xu et al. [17] proposed a wall-climbing drilling robot with an expansion self-positioning mechanism and joint torque compensation to align the drill spindle to the surface normal in confined geometry, and Yang et al. [18] addressed the same challenge on large curved components using an adaptive Principal Component Analysis (PCA)-based vision-driven normal estimation. Madani et al. [19] augmented a KUKA LBR iiwa 7 cobot with adaptive admittance control and haptic guidance, allowing operator–robot cooperation to ensure correct alignment and stability on curved surfaces, and Lee et al. [20] further enhanced drilling quality through combined implicit force and position control to counteract tool deflection and parasitic forces. Pereira et al. [21] optimized a flexible industrial robotic drilling system using a KUKA KR-16 with a high-speed spindle for machining aluminum aerospace alloys, focusing on reducing burr formation and surface roughness through tuning of feed rate, spindle speed, and pecking cycles. Miyake & Kondo’s constant-load feeding method [22], developed for cobot-based drilling, reduced vibration and exit burrs even when clamping or rigid fixturing was limited. Guo et al. [23] developed a mobile robot for fuselage drilling or riveting, which navigates along the assembly using onboard laser sensors and artificial landmarks to maintain positioning accuracy standards. In contrast to the traditional drilling methods, these robotic drilling methods provide higher flexibility to the aerospace production environments.

However, common automated drilling systems come with significant drawbacks: they are costly, mechanically complex, maintenance-heavy, occupy large footprints, require strict safety barriers, and often involve lengthy commissioning cycles. Consequently, their application is largely limited to high-volume production lines and remains inaccessible for smaller or reconfigurable aerospace operations. Safety concerns further compound these limitations, especially in collaborative environments where human–robot interaction is expected. Industrial robots, with their high inertia and rigidity, pose considerable risks to operators. To address this, researchers have proposed safety-oriented solutions, such as adding passive compliance to robot joints [24], end-effector airbags [25], and low-impedance actuation strategies [26], all of which reduce collision hazards and improve operator safety. The use of low-density and lightweight structural materials, such as aluminum and carbon fiber, has also been shown to reduce inertia and create safer, more adaptable interaction environments [27,28].

Collaborative robots (cobots) have emerged as a promising alternative. With inherent safety features and lower cost, they enable flexible deployment in shared workspaces. Yet, their limited payload capacity prevents them from generating the large clamping forces required for aerospace drilling tasks. This creates a gap between the performance offered by conventional drilling robots and the flexibility needed in collaborative environments. To address these limitations, various strategies have been explored in the literature. Cobot payload is enhanced using optimized trajectory planning and balancing the gravitational load without a substantial change in structural mass [29,30]. Likewise, advancements in flexible-link manipulators and variable-stiffness designs have been introduced to enhance the payload-to-weight ratio, resulting in improved energy efficiency and motion agility without compromising accuracy [28,31]. Another notable approach, the Independent Load and Measurement Arm (ILAMA), separates the sensing and load-bearing functions, allowing collaborative robots to handle higher payloads while maintaining the precision required for aerospace manufacturing tasks [27].

These methods have proven effective only in increasing the dynamic payload of the cobot to some extend during moving through defined paths; however, the gains remain insufficient to meet the static clamping and drilling-phase requirements of aerospace applications. Beyond generating the required clamping force at the tool–workpiece interface, a reliable cobot-based drilling system must also deliver sub-millimeter positioning accuracy, maintain precise surface-normal alignment, and suppress process vibration during drilling, approaching the performance typically achieved by large, high-footprint automated drilling systems such as the KUKA KR210. Achieving this combination of force capability, accuracy, and stability within a compact, safe, and cost-efficient platform remains an open challenge. Consequently, there is a strong motivation for developing an adaptable End-of-Arm Tool (EoAT) capable of providing localized force amplification and metrology-grade positioning while preserving the inherent safety and flexibility of cobot deployments in collaborative aerospace drilling.

There have been attempts to propose EoAT concepts to bridge the gap between aerospace drilling requirements and the limited force capacity of collaborative robots. Boeing’s Flexible Drilling System (FDS) demonstrated a self-attaching vacuum-based drill unit mounted on a cobot, capable of bracing itself against the aircraft skin for improved drilling stability. While this approach enabled higher thrust during drilling, its limited normality correction mechanism may prove inadequate for complex, double-curvature fuselage surfaces where precise alignment with the local surface normal is critical [32]. Xu et al. proposed a self-locking end-of-arm clamping device to counteract drilling forces and improve stiffness by anchoring the tool to the workpiece [33]. However, the system relied on peripheral support infrastructure and a guided drilling template. it offered limited surface compliance, requiring precise setup for each drilling location, which prolongs drilling cycles. The system was designed for confined spaces and was not integrated with an independent collaborative robot and therefore did not fully leverage the benefits of safe human–robot interaction. Kang et al. proposed a serial-parallel hybrid drilling robot that moves along the flexible circular track attached to the fuselage skin, which is accompanied by a high-fidelity digital twin [34]. The need to install a large peripheral structure around the fuselage increases system complexity and prolongs the overall drilling cycle. Design of an End-of-Arm Tool mounted on a cobot may offer shorter drilling times while enabling close human–robot collaboration due to its smaller footprint. Another drawback of the system designed by Kang et al. [34] is that the normality correction mechanism they adopted relies on gauge readings and multiple computations within a complex model, which may introduce alignment errors and reduce robustness in real-world settings.

While improving automated solutions is essential, increasing aerospace drilling performance also requires an intelligent and flexible human–robot interface to easily plan and modify tasks and control the system. One promising approach is to integrate the robot, the workpiece, and the drilling process within a cyber–physical system (CPS) that leverages digital twin (DT) technology through an interactive user interface with visualization and simulation capabilities. This approach will enable operators with limited programming skills to seamlessly define drilling tasks and monitor the process carefully. The graphical user interface (GUI) of the CPS can be designed to enable the end-user to define drilling tasks for different variations of the robot and workpiece. Most DTs developed for the manipulators in the literature focus on mirroring the physical entity, merely allowing monitoring of the process and real-time simulations, with a minimal level of interactivity for the user. Moreover, those with a user interface are mainly for manipulating the robot joints manually from the GUI, and very few studies focus on interactively defining tasks using the workpiece model rendered in the visualization panel. Furthermore, most of the existing CPS frameworks are developed exclusively for singular tasks and lack modularity in development to extend them to other applications. In this work, we devised a modular CPS with an interactive GUI and a real-time DT of the proposed drilling cobot. In this CPS, the operator can select drilling holes and plan tasks directly from the configured workpiece geometry in the render panel, and the model of the robot is synchronized with the physical counterpart. The modularity of this CPS allows for straightforward extension of the current development to new clients with applications other than drilling.

CPS and DTs have gained attention recently for mobile robots [35,36] and manipulator applications. To mention a few related works, Ruiz Garcia et al. [37] presented a CPS for collaborative assembly in which an interface was designed to enable the operator to coordinate an industrial manipulator. Automated drilling processes may also take advantage of an interactive CPS that facilitates operation and supervision of the process [38]. Farhadi et al. [39] developed a DT for a robotic drilling manipulator, but the DT primarily served for real-time visualization, monitoring, and prediction, without meaningful user interaction. Guerra Zubiaga et al. [40] adopted a DT for monitoring and regulating the drilling speed of a FANUC robot. Their use of DT and the proposed Node-RED dashboard still had limited operator interactivity and focused on managing the drilling speed, analyzing joint influences using the design of experiments methodology in the virtual environment, and predicting the implementation of the drilling process, while lacking a reliable and user-friendly interface for task planning by end-users in real-time. Xiang et al. [41] developed a web-based DT platform for industrial robot manufacturing, with process visualization and operation, but relied on Unity 3D, which is computationally heavy for low-end processors. Moreover, their GUI only allowed high-level control, and interaction with geometric entities in the visualization panel was not available. The Robot Operating System (ROS) can be a reliable foundation for CPS due to its node communication feature, enabling modularity. Malvido Fresnillo et al. [42] devised a reconfigurable ROS-based user interface for four industrial use cases. Kuts et al. [43] proposed a ROS-based DT user interface compatible with both manipulators and mobile robots. However, yet again, their GUI lacked interactivity with the workpiece, and also, it was not validated or synchronized with its counterpart’s physical twin. From the literature, it can be concluded that DTs have often been underutilized, being employed mostly for forecasting the movements and supervising the process [44].

In this study, the Advanced Collaborative Multifunctional End-Effector (ACME) is introduced as a lightweight robotic drilling tool for integration with cobots. ACME integrates a passive self-normalization mechanism for angular alignment, vacuum-assisted clamping, and a compact, lightweight, and safe form factor compatible with collaborative use. This End-of-Arm Tool enables cobots to perform aerospace-grade jig-less drilling operations that were previously infeasible for them. In parallel, this work incorporates the drilling process into a CPS with an interactive DT. Unlike conventional DT implementations that primarily provide passive visualization, the proposed CPS allows operators to configure workpieces interactively, select drilling locations, and monitor execution directly from a graphical interface. This reduces programming barriers, improves accessibility, and enables reconfigurable deployment across diverse aerospace parts. The main contributions of this work are to:

- Design, prototype, and analyze a lightweight, cobot-compatible drilling end-effector that enables a cobot to apply aerospace-grade clamping forces, beyond its inherent capability, while maintaining high positioning accuracy on flat, simple-, and double-curvature panels. This allows the system to approach the performance of conventional large-footprint automated drilling systems while reducing cost and improving safety for human-proximate operation.
- Introduce a passive self-normalization mechanism for alignment correction on double-curvature surfaces, eliminating the need for additional actuators and feedback sensors, thereby reducing system complexity and weight, while maintaining accurate TCP position/orientation under structural deflection.
- Despite other collaborative solutions, the proposed system is the only fully automated, jig-less drilling solution that achieves a cycle time comparable to large, non-collaborative robotic platforms.
- Develop and implement a CPS with an interactive DT to enable intuitive task planning and monitoring.
- Validate drilling performance through experimental trials on representative aerospace workpieces.

Meeting these requirements necessitates innovative mechanical design and careful integration with collaborative robotic platforms. ACME was developed in this context to demonstrate that aerospace-level drilling performance can be achieved with a lightweight cobot-compatible solution.

## 2. ACME End-of-Arm Drilling Tool

ACME was conceived to bring aerospace-grade drilling performance to collaborative cells by pairing a lightweight End-of-Arm drilling Tool with cobots and incorporating mechanisms that guarantee surface alignment and clamping on double-curvature panels. Developing an automated drilling end-effector for aerospace manufacturing requires meeting a set of strict criteria to guarantee reliable operation in production environments. Large industrial drilling systems (e.g., KUKA 210) require fenced-off areas, limiting their use around humans. ACME was therefore intended as a compact module compatible with cobots and their built-in safety features to provide the essential capabilities of conventional drilling systems while maintaining a small footprint and removing the need for physical barriers, enabling safe and efficient human–robot collaboration.

Effective drilling of stacked composite–metal assemblies demands strong layer compression to avoid gaps, burrs, and delamination; prior work indicates that at least 500 N is needed even for two 2 mm aluminum sheets. Cobots cannot inherently provide sufficient clamping forces required for jig-less aerospace drilling. Existing cobotic solutions (ARM, DLR, Safran) are therefore very limited in their work scope and require special tooling. Consequently, in ACME design we were tasked with providing higher clamping-force capability.

Integrating ACME with a cobot provides a cost-effective, compact, and safe alternative to traditional automated drilling systems. Large industrial robots (e.g., KUKA 210) paired with proprietary end-effectors and software can exceed 2 million CAD$, require extensive floor space, and rely on fenced enclosures. In contrast, cobots like the UR10e are far more affordable and easier to deploy, and with ACME’s low production cost target, the overall system should stay accessible for deploying multiple units simultaneously.

Most high-end cobots, such as the UR20, Fanuc CRX-25iA, and KUKA LBR iisy, support only 15–30 kg of payload, making lightweight design essential. Traditional aerospace drilling end-effectors often exceed 100 kg. ACME’s weight was therefore set to remain within 15–20 kg, fully compatible with cobots for safe, efficient drilling. Aerospace drilling demands high precision, typically <0.2 mm positioning error and ±0.5° normality, even on double-curvature surfaces where the local normal changes continuously. Conventional automated systems can meet these tolerances but rely on large robots and active normalization units that increase cost, weight, and susceptibility to backlash or instability. ACME addresses these challenges with a lightweight passive self-normalizing mechanism that preserves drill alignment without motorized correction, reducing complexity and improving reliability. While the prototype aims to approach industry-level accuracy, some deviation from full production standards will be considered acceptable at this stage.

Beyond force, weight, and accuracy, ACME was also expected to provide a sufficiently large local workspace to drill multiple holes without frequent repositioning, enabling short cycle times and higher productivity compared to existing cobotic tools that require relocation for each hole. Its cobot-mounted design eliminates bulky fixtures and setup overhead, avoiding the repeated installation of internal or external alignment structures common in state-of-the-art systems. Table 1 summarizes the target specifications, both based on the ideal aerospace standards and the prototype design goals, and compares ACME’s design objectives with available robotic drilling solutions. This comparison is intended only as a high-level overview based on publicly available data, as direct measurement of these systems was not feasible. The listed specifications serve as benchmarks guiding ACME’s development and will provide reference criteria for validating the system’s performance once built.

**Figure 2:**
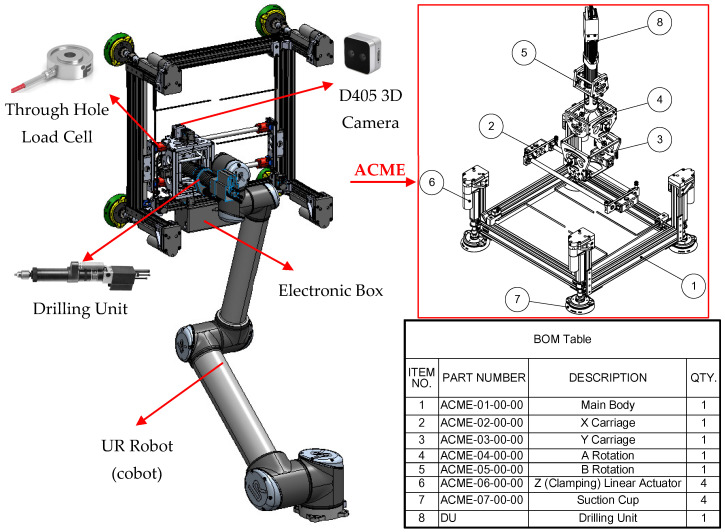
ACME is mounted on a UR robot to extend its reach and take advantage of the cobot’s safe workspace, while it enables generating the required clamping force through suction cups.

Figure 2 shows ACME mounted on a UR robot. The tool weight is engineered to remain within typical cobot payload capacities while providing passive self-normalization and integrated vacuum-assisted clamping to stabilize the end-effector on multi-material stacks during drilling. As illustrated in Figure 2, the ACME features a compact architecture providing five active degrees of freedom (5-DoF), achieved through the combination of three translational motions along the X, Y, and Z axes and two rotational movements about the A and B axes. This arrangement allows accurate planar positioning as well as orientation control to ensure the drilling tool remains normal to curved surfaces. The lightweight structural frame acts as the supporting chassis, hosting the linear stages, rotary joints, sensing units, and the drilling module itself. Integrated components include a force-sensing transducer for real-time load feedback, vision sensors for part localization, and suction-based anchors that provide stable surface adhesion and clamping. When coupled with a collaborative robot, ACME extends the cobot’s operational versatility, taking advantage of its safe human–robot interaction features, while overcoming its payload limitations through direct surface mounting.

**Figure 3:**
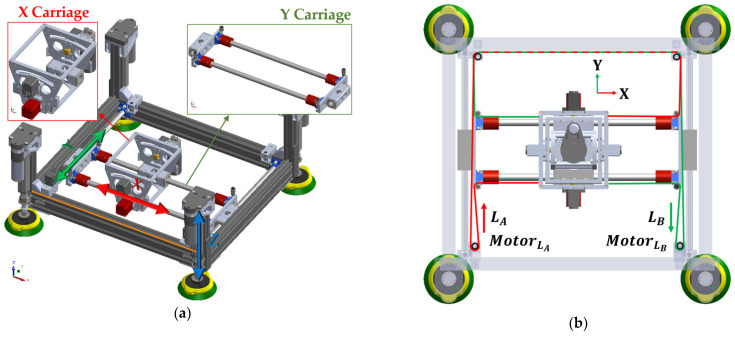
(**a**) shows the X-Y-Z motions that are provided by two carriages (X carriage/axis in red and Y in green) and four synchronized linear actuators (Z axis in Blue), and (**b**) shows the alignment of the CoreXY mechanism for the XY motion.

The main structural element of ACME is a rigid aluminum frame designed and optimized to sustain the drilling and clamping forces characteristic of aerospace applications. As shown in Figure 3a, four linear actuators equipped with suction cups provide Z-axis motion and clamping by driving the frame toward or away from the workpiece. For planar movement, Figure 3b illustrates a CoreXY-based carriage system that travels along belt-driven rails, ensuring smooth motion with minimal backlash. The CoreXY configuration utilizes two fixed motors connected by crossed belts to generate coordinated X–Y translation of the drilling unit. This architecture minimizes moving mass, enhances stiffness, and enables fast, repeatable positioning across the workspace without frequent re-attachment or complex robot maneuvers. Mounting the motors on the stationary frame reduces inertia, while the belt-driven design improves accuracy and reliability within a compact, easily maintainable structure.

**Figure 4:**
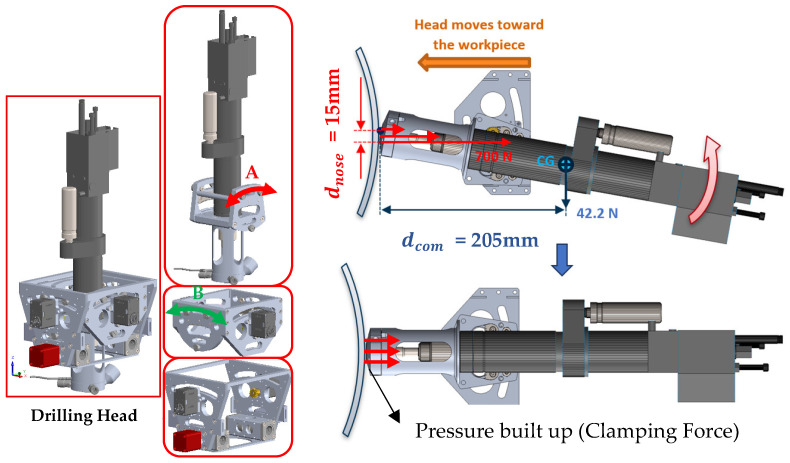
Automatic self-alignment of the drilling nose to maintain perpendicularity on double curvature surfaces. As the nose contacts the surface, the distribution of the load (represented with red arrows) aligns it to be normal to the surface. Once aligned, the uniform force distribution keeps the nose always normal to the surface during drilling.

At the drilling interface, two rotational degrees of freedom, pitch and yaw (axes A and B), allow the end-effector to adapt to local surface curvatures. These rotations are implemented through a compact gimbal mechanism integrated into the X-axis carriage. Depending on system requirements, the joints can be either actively actuated by servo motors for precise orientation control or passively adjusted through mechanical linkages responding to contact forces. This configuration ensures the drill bit remains perpendicular to curved panels, thereby enhancing hole geometry and minimizing taper or ovality defects. Each rotational joint provides approximately ±10° of angular adjustment, sufficient to maintain normal alignment over double-curved aerospace components. As shown in Figure 4, in the passive mode, the reaction torque from the clamping force counteracts the gravitational moment on the drilling head, allowing it to self-align orthogonally with the surface.

Recent work in underactuated manipulation has shown that embedding mechanical intelligence that allows hardware to conform naturally to surfaces improves robustness and reliability while reducing control complexity in unstructured industrial settings. Passive adaptability and constraint exploitation, as demonstrated by Turco et al. [47] and Eshraghi et al. [48], enhance end-effector stability and fault tolerance while simplifying control. Building on this principle, ACME’s passive self-normalizing drilling nose leverages geometric constraint exploitation and passive torque balancing to achieve robust surface alignment with minimal sensing, making it inherently suited for high-reliability aerospace manufacturing environments.

The geometry and force requirements of the passive self-normalizing drilling nose were determined by analyzing the moment balance about the mechanism’s center of rotation. For passive alignment to occur, the reaction torque generated by the clamping force at the drilling nose must overcome the gravitational moment acting on the drilling unit in its worst-case orientation. Two primary torques act around the pivot point: (1) the gravitational moment tending to rotate the unit away from the surface normal, and (2) the reaction moment produced by the clamping force applied through the drilling nose.

(1) $$M_{gravity}=m_{DU}\cdot g\cdot d_{com}$$

(2) $$M_{clamp}=F_{clmap}\cdot d_{nose}$$

Here, m=4.22 kg is the mass of the drilling unit, $g=9.81{\;m/s}^{2}$, $d_{com}=205\;\mathrm{mm}$ is the horizontal offset between the pivot and the center of mass, and $d_{nose}=15\;\mathrm{mm}$ is the moment arm from the pivot to the clamping reaction point on the nose. Passive normalization initiates when $M_{clamp}\geq M_{gravity}$.

Solving for the minimum required clamping force yields

(3) $$F_{min}=\frac{m\cdot g\cdot d_{com}}{d_{nose}}=565.77\;N$$

This analysis informed the geometric design and tolerance selection of the nose and pivot assembly. The suction cups and clamping hardware were selected to exceed this minimum required force with a substantial safety margin, ensuring consistent and robust passive alignment. It is important to note that the calculated $F_{\min}$ represents the worst-case force needed to initiate rotation; in many orientations of the ACME, passive normalization begins at lower clamping forces. Furthermore, as can be seen, this passive self-normalization mechanism has a lower level of mathematical complexity compared to state-of-the-art active normalization systems, such as the one developed by Kang et al. [34].

**Figure 5:**
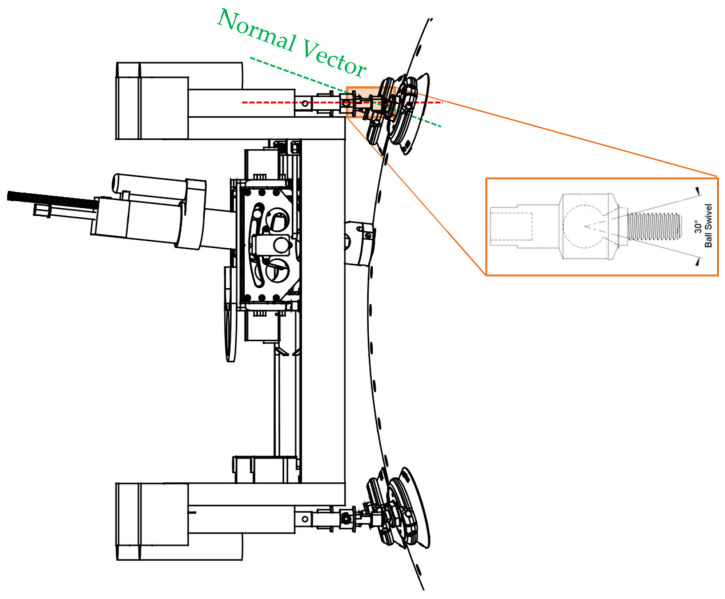
Ball joints at the end of linear actuators and before the suction cups help with alignment on curved surfaces.

As illustrated in Figure 5, each linear actuator of ACME is connected through a ball joint providing ±30° of angular compliance, enabling each suction cup to align and adhere to curved surfaces with radii down to approximately 1.5 m, as well as flat panels. Additionally, they are equipped with an independent vacuum security valve that automatically isolates a cup in the event of partial or complete loss of contact, thereby preventing pressure loss across the entire suction network and maintaining stable adhesion. The actuators are designed to withstand gravitational loads and to operate reliably in vertical orientations, providing sufficient force and speed to overcome inertial effects during surface alignment and drilling operations. The remainder of the drilling process remains identical regardless of whether the tool is mounted on flat or curved surfaces, or in horizontal or vertical orientations.

**Figure 6:**
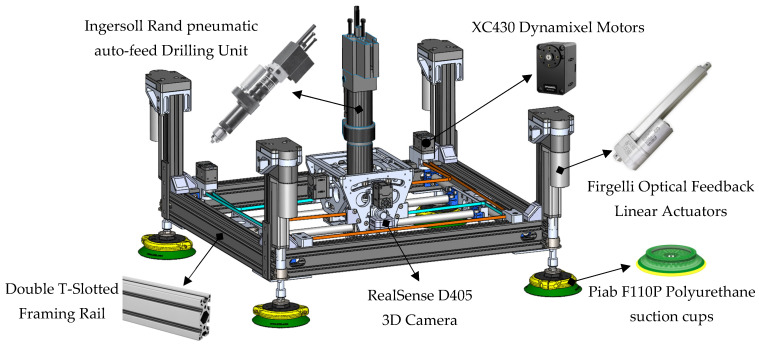
Main components selected for the prototype.

The prototype is primarily composed of off-the-shelf components, which substantially simplifies development and reduces cost compared to custom-fabricated assemblies (see Figure 6). The main structure uses 6560-T6 hollow aluminum T-slotted framing, chosen for its high stiffness-to-weight ratio and its modularity. The open-slot geometry allows rapid reconfiguration and the addition or removal of sensors and subassemblies without redesign. For surface attachment, four Piab F110P polyurethane suction cups were selected. Each cup provides a maximum normal lifting force of 591 N and a permissible shear load of 617 N at 90 psi, resulting in a combined capacity of 2364 N in lifting and 2468 N in shear, values that exceed the requirements for conventional aerospace-grade materials and the expected operational loads. The cups are actuated by four Firgelli optical-feedback linear actuators, each capable of producing 890 N over a 3-inch stroke at 6–10 mm/s. Together, they can generate up to 3560 N of clamping force, exceeding the required level, while remaining the most compact off-the-shelf actuators available. In practice, the limiting factor in clamping capability is the suction-cup lifting capacity rather than the actuators themselves; however, the effective clamping capacity remains well above the typical 50–150 kg required for aerospace stack-ups. These actuators also provide encoder feedback for position sensing and reliable closed-loop control. X–Y translation and A–B tilting are driven by high-power-density Dynamixel XC430 servomotors equipped with 4096-pulse/rev contactless absolute encoders. With the implemented gearing, the motors deliver up to 250 N of in-plane force at 3.7 m/min for the X–Y axes and up to 58 N·m at 5.5 rev/min for the A–B axes, as summarized in Table 2. Mounted on the A–B assembly is a 0.50 HP Ingersoll Rand pneumatic auto-feed drilling unit (DU), widely used in aerospace manufacturing. Operating at 90 psi and 5000 RPM (83 Hz), it provides a 32 mm feed stroke and sufficient torque for multi-layer aluminum stacks. A concentric through-bore load cell continuously measures thrust and clamping forces, enabling closed-loop regulation and consistent hole quality. The prototype weighs approximately 18 kg, which, although slightly above our initial ideal target in Table 1, it is still within the range of payload of cobots in general.

The “Multifunctional” designation in ACME reflects its modular end-effector architecture. Although the current prototype integrates a drilling unit, the DU can be readily exchanged for advanced drilling units (ADUs), fastener-insertion heads, percussive tools, or inspection and metrology devices without altering the core mechanisms. Regardless of the attached tool, ACME preserves its capabilities for accurate positioning, automatic normal alignment, and controlled clamping. Overall, the assembly remains lightweight for collaborative-robot operation while meeting the rigidity, precision, and integration requirements of aerospace drilling and related manufacturing tasks.

**Figure 7:**
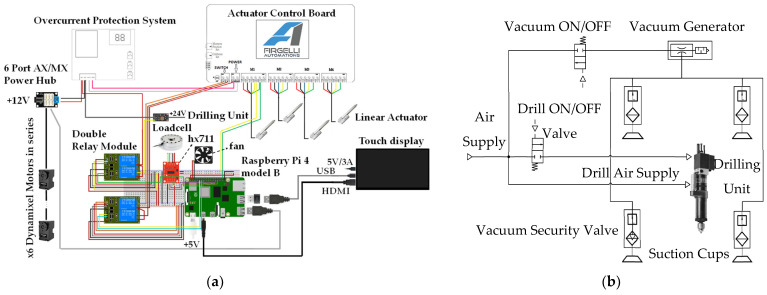
(**a**) Connection layout of mechatronics components. (**b**) Layout of the pneumatic system.

ACME combines integrated electronic and pneumatic architectures to support actuation, sensing, and control functionalities. A Raspberry Pi 4 serves as the main processing unit, managing sensor acquisition, control, and visualization. Through its GPIO interface, the board enables direct communication with peripheral components, while an actuator controller synchronizes the motion of the four Z-axis linear actuators using real-time feedback from the embedded load cell to maintain a consistent clamping force. The system also includes an overcurrent protection circuit that functions as an emergency cut-off for safety. Serial communication lines govern relays and solenoid valves, and a touchscreen interface provides real-time monitoring and user control. As illustrated in Figure 7a, the electronic layout is organized with isolated wiring channels to minimize electrical noise and enhance system reliability. The pneumatic module, shown in Figure 7b, employs a Venturi vacuum generator powered by compressed air to anchor the device to the work surface via suction cups equipped with mechanical cleats. Vacuum retention valves safeguard against accidental detachment, while solenoid valves regulate airflow to the drilling unit and vacuum generator, maintaining suction stability during operation and enabling quick release for repositioning. Collectively, these embedded electronic and pneumatic subsystems ensure precise drilling, stable clamping, and safe functionality, effectively compensating for the limited payload capacity of collaborative robots.

## 3. Kinematics and Dynamics Modeling

Accurate kinematic and dynamic representations of the Advanced Collaborative Multifunctional End-Effector (ACME) are essential to ensure repeatable, high-precision drilling performance under the stringent tolerances demanded in aerospace production. In contrast to conventional serial manipulators composed solely of revolute joints, ACME employs a hybrid configuration comprising three translational (X, Y, Z) and two rotational (A, B) degrees of freedom. This arrangement enables simultaneous control of position and orientation, allowing the drilling axis to remain normal to curved or contoured surfaces.

**Figure 8:**
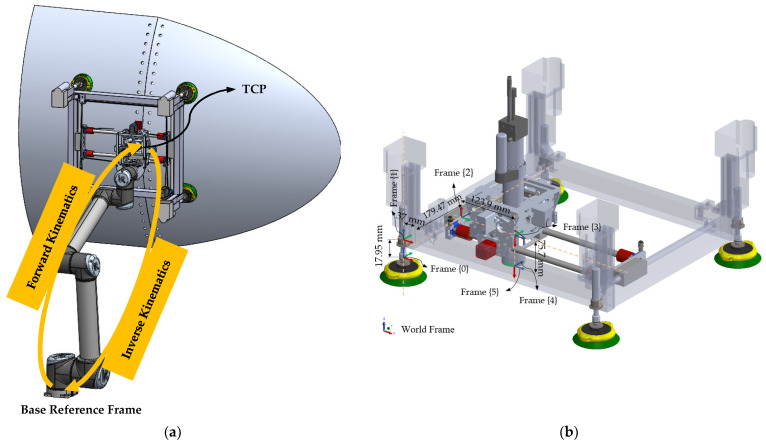
(**a**) Forward and inverse kinematics relate the TCP pose to the base frame, enabling mapping in both directions. (**b**) The Cartesian coordinate frames definitions for ACME 5-DoF are shown, which give the associated DH parameters.

The system’s kinematic structure is formulated using the modified Denavit–Hartenberg (DH) convention [49], which provides a systematic approach for assigning local coordinate frames along the joint chain. Each frame is characterized by four geometric parameters: link length $a_{i}$, link offset $d_{i}$, link twist $\alpha_{i}$, and joint angle $\theta_{i}$. As illustrated in Figure 8b, the base frame {0} is defined at the midpoint between the suction-cup ball joints, while frames {1} through {3} correspond to the Z-, Y-, and X-axis prismatic translations. The final frames, {4} and {5}, represent the B- and A-axis rotations positioned at the drilling nose, defining the tool’s orientation relative to the work surface.

**ACME Forward Pose Kinematics (FPK).** The forward pose kinematics (FPK) of a serial manipulator determines the spatial pose—namely, the position and orientation—of the end-effector from a given set of joint variables. In serial robotic architectures, this computation is typically direct and methodical, relying on the Denavit–Hartenberg (DH) parameters (summarized in Table 3) that define each link’s geometry. By substituting these parameters sequentially into the standard homogeneous transformation formulation, the relationship between successive coordinate frames {i−1} and {i} can be systematically obtained, as presented in [49].

(4) Tii−1=cθi−sθi0ai−1sθicαi−1cθicαi−1−sαi−1−disαi−1sθisαi−1cθisαi−1cαi−1dicαi−10001=Rii−1Pii−10001

where the following abbreviations were used: $c\theta_{i}=\cos\theta_{i}$, $s\theta_{i}=\sin\theta_{i}$, $c\alpha_{i}=\cos\alpha_{i}$, and $s\alpha_{i}=\sin\alpha_{i}$.

This homogeneous transformation matrix provides the foundation for describing both geometric and kinematic dependencies throughout the mechanism, ultimately enabling the derivation of the complete transformation from the base frame to the tool center point (TCP). For the ACME system, which incorporates five active degrees of freedom in a serial chain, the FPK can be expressed as follows:

Given a set of known ACME joint variables $\left(d_{1},d_{2},d_{3},\theta_{4},\theta_{5}\right)$ and the cobot joint variables $\left(\theta_{c,1},\theta_{c,2},\theta_{c,3},\theta_{c,4},\theta_{c,5},\theta_{c,6}\right)$, the aim is to achieve the transformation matrices TACME50 and TCobotTCPB, where

- $\left(d_{1},d_{2},d_{3}\right)$ denote prismatic displacements along the translational axes, and ${(\theta }_{4},\theta_{5})$ represent the rotational joints of ACME;
- {TCP} corresponds to the tool-center-point frame, and {B} denotes the cobot’s fixed base frame (see Figure 8a).

All coordinate frames follow a right-handed Cartesian convention, indicated by curly braces { }. The ACME mechanism includes six defined reference frames, {0} through {5}, with {0} serving as the base and {1–5} corresponding to individual actuated joints. By substituting each DH parameter set from Table 3 into the general homogeneous transformation formulation (Equation (5), five successive transformation matrices are obtained, collectively defining the full spatial pose of the end-effector.

(5) T10=10000100001d1+17.950001, T21=01037001d2+179.4710000001T32=−1000001d3+123.901000001, T43=cos(θ4−pi/2)−sin(θ4−pi/2)075.70010−sin(θ4−pi/2)−cos(θ4−pi/2)000001T54=cos(θ5+pi/2)−sin(θ5+pi/2)0000−10sin(θ5+pi/2)cos(θ5+pi/2)000001

The forward pose kinematics (FPK) of the ACME is obtained by sequentially multiplying the homogeneous transformation matrices associated with each joint, followed by the cobot’s transformation matrix:

(6) TTCPB=TFlangeBT0FlangeT50d1,d2,d3,θ4,θ5TTCP5

The transformation between frames {0} and {5} for ACME is given by:

(7) T50d1,d2,d3,θ4,θ5=−c4s5−c4c5s4d3+160.9c5−s50d2+179.47s4s5c5s4c4d1−57.750001

where $c_{i}=\cos\theta_{i}$, $s_{i}=\sin\theta_{i}$ for i=4, 5, TFlangeB is the homogeneous transformation matrix of the cobot, depending on its joint angles $\left(\theta_{c,1},\theta_{c,2},\theta_{c,3},\theta_{c,4},\theta_{c,5},\theta_{c,6}\right)$, T0Flange is a fixed transformation matrix describing the mounting offset between the cobot flange and the ACME base, T50d1,d2,d3,θ4,θ5 represents the ACME kinematic chain, and TTCP5 defines the fixed pose of the tool center point (TCP), which coincides with frame {5}.

In the ACME configuration, frames {2} and {3} correspond to the CoreXY planar stage, while frames {4} and {5} represent the rotational A and B axes located at the drill nose. For the CoreXY mechanism, the Cartesian displacements of the end-effector are expressed in terms of the belt lengths of Motors A and B as follows:

(8) $$d_{3}=X=1/2(L_{A}+L_{B})$$

(9) $$d_{2}=Y=1/2(L_{B}-L_{A})$$

where $L_{A}$ and $L_{B}$ are the linear displacements of Motors A and B, respectively. Conversely, when the desired TCP coordinates (X,Y) are known, the required motor displacements can be determined as:

(10) $$L_{A}=-X+Y=-d_{3}+d_{2}$$

(11) $$L_{B}=X+Y=d_{3}+d_{2}$$

Accordingly, the transformation matrix of ACME can also be represented in terms of motor displacements $L_{A}$ and $L_{B}$:

(12) T50d1,LA,LB,θ4,θ5==−c4s5−c4c5s412LA+LB+160.9c5−s5012LB−LA+179.47s4s5c5s4c4d1−57.750001

**ACME Inverse Pose Kinematics (IPK).** IPK aims to identify the set of joint coordinates required to place and orient the tool center point (TCP) at a specific target pose. According to Pieper’s criterion, a serial manipulator with six degrees of freedom (6-DoF) can be solved analytically when three successive rotational axes intersect at a single point—forming what is known as a spherical wrist. For the ACME architecture, this requirement is met by interpreting the TCP frame as an additional, virtual sixth joint coinciding with the A and B rotational frames {4} and {5}. Because the transformation TTCP5 is defined as an identity; this assumption effectively yields the same kinematic characteristics as a spherical wrist, permitting an analytical solution for the IPK.

Under this configuration, and given the kinematic configuration pictured in Figure 8b, a closed-form and computationally efficient formulation can be developed to determine the ACME joint states corresponding to any desired end-effector pose. The inverse kinematic problem is thus defined as follows:

- Given: Fixed Denavit–Hartenberg parameters (Table 3) and a target TCP pose described by the homogeneous transformation matrix TTCP0.
- Find: The joint variables $\left(d_{1},L_{A},L_{B},\theta_{4},\theta_{5},\theta_{TCP}\right)$ that achieve this pose.

In practice, a fixed vision sensor located on ACME is used to locate and register the workpiece to the base of ACME (frame {0}), and hole patterns are provided as a general transformation matrix TTCP0. The analytical solution begins by isolating the intermediate transformation matrix T50 from the known overall pose using:

(13) TTCPB=T0BT50TTCP5

(14) T50=T0B−1TTCPBTTCP5−1

As discussed, TTCP5 is designed to be an identity matrix and is not based on the Denavit–Hartenberg parameter table. The identification and localization of the workpiece occur after the cobot places ACME on the surface, and it is secured and mounted to the surface through suction cups; so, the position and orientation of the robot (T0B) would be known. Thus, IPK for ACME is directly derived from FPK, which simplifies the IPK formulation, as translational and rotational variables can be treated independently.

(15) T50d1,LA,LB,θ4,θ5=r11r12r13x50r21r22r23y50r31r32r33z500001=−c4s5−c4c5s412LA+LB+160.9c5−s5012LB−LA+179.47s4s5c5s4c4d1−57.750001

From this, the joint variables can be extracted as:

(16) d1=z50+57.75

(17) x50=0.5×LA+LB+160.9

(18) y50=0.5×LB−LA+179.47

Rearranging Equations (14) and (15) gives the linear motor displacements:

(19) LA+LB=2×x50−321.8LB−LA=2×y50−358.94→LB=x50+y50−340.37LA=y50−y50+18.57

Finally, the orientation angles $\theta_{4}$ and $\theta_{5}$ are determined using the quadrant-aware inverse tangent function atan2, ensuring unique solutions:

(20) $$\theta_{4}=atan2\left(r_{13},r_{33}\right)=atan2\left(s_{4},c_{4}\right)$$

(21) $$\theta_{5}=atan2\left({-r}_{22},r_{21}\right)=atan2\left(s_{5},c_{5}\right)$$

This formulation provides a closed-form and computationally efficient IPK solution for the ACME mechanism, enabling accurate pose estimation and joint control during drilling operations.

**Dynamics and numerical implementation.** Beyond kinematics, the dynamic model of ACME was developed to predict actuator efforts under gravity and drilling loads and to evaluate transient positional displacements and vibrations at the end-effector. The dynamic evaluation was conducted under the clamped configuration, representing the drilling condition in which the end-effector is fully constrained to the workpiece. In this state, the dominant dynamic behavior arises from the structural properties of the plant, including link inertias, joint and transmission flexibility, and fixture compliance. Therefore, the controller was excluded from the dynamic and vibration analysis to preserve the intrinsic mechanical characteristics of the structure; the control loops act primarily as position-holding mechanisms and do not influence the natural modes of the system in this configuration. The plant dynamics were formulated in joint space using a compact representation suitable for numerical implementation in MATLAB Simulink. Let $q=\;{\left[d_{1},d_{2},d_{3},\theta_{4},\theta_{5}\right]}^{T}$ and $q_{m}$ the corresponding motor-side coordinates. The coupled motor–link system is expressed as:

(22) $$M_{l}\left(q\right)\ddot{q}+C\left(q,\dot{q}\right)\dot{q}+g\left(q\right)+D_{l}\dot{q}+K\left(q-q_{m}\right)\;=\tau_{ext},$$

(23) $$J_{m}{\ddot{q}}_{mi}+C_{m}{\dot{q}}_{mi}+K\;(q_{mi}-q_{i})=\tau_{m}(t)$$

Here, M, C, g, and $D_{l}$ represent the link-side inertia, Coriolis/centrifugal, gravity, and damping terms; $J_{m}$ and $C_{m}$ are diagonal matrices of motor inertia and damping; and K is block-diagonal, containing the linear stiffness for the prismatic axes and torsional stiffness for the revolute axes. The vector $\tau_{ext}$ includes all external wrenches mapped into joint space through the geometric Jacobian.

The compliance of the suction-cup fixture was modeled through four planar joints, each allowing small deflections along local X and Y directions and rotation about the Z-axis. The nominal attachment configuration of each cup corresponds to its equilibrium position, and the small deflections are defined with respect to this state. The local restoring wrench of cup j is

(24) $$w_{j}^{loc}=K_{p}\eta_{j}+D_{p}{\dot{\eta }}_{j},$$

where $\eta_{j}=[\delta x_{j},\;\delta y_{j},\;\delta \theta_{z,j}{]}^{T}$ is the local displacement vector, and $K_{p}=diag(K_{x},K_{y},K_{\theta })$ and $D_{p}=diag(D_{x},D_{y},D_{\theta })$ are the stiffness and damping coefficients. The corresponding wrench at the tool-center point (TCP) is obtained by rigidly mapping the local response of each cup to the global frame:

(25) $$w_{\mathrm{cups}}=\sum_{j=1}^{4}A_{j}^{⊤}w_{j}^{loc},$$

where $A_{j}$ represents the transformation matrix between the TCP and the $j^{\mathrm{th}}$ suction cup. Axial compression effects along the Z-direction are modeled separately as additional scalar spring–damper elements for each cup and linear actuator. The total external generalized load applied to the mechanism is therefore expressed as

(26) $$\tau_{ext}=J^{T}(q)(w_{drill}+w_{cups}),$$

where $w_{drill}$ denotes the drilling thrust and torque applied at the TCP.

**Figure 9:**
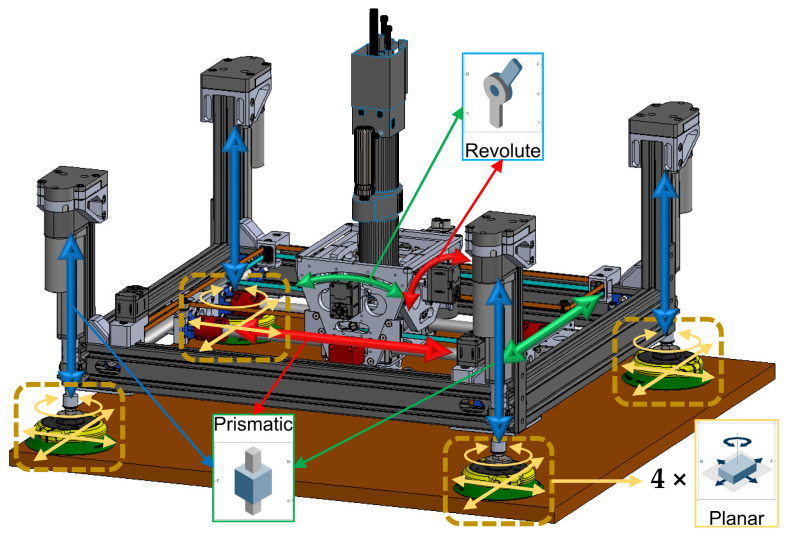
A dynamic representation of the system includes multiple compliant joints.

The model encompasses all degrees of freedom in the mechanism: four compliant internal joints (two X-Y prismatic and two A-B rotational), twelve compliant degrees of freedom introduced by the four suction-cup attachments (each suction cup modeled as a planar joint), and four vertical (along Z) DoFs corresponding to the suction-cup deformation and linear-actuator compliance along the Z-axis. Altogether, the system exhibits twenty coupled degrees of freedom, capturing both the rigid and compliant behaviors of ACME, while considering the main structure (connecting frames and components) as rigid (Figure 9). The formulation of Equations (22)–(26) enables efficient numerical integration and provides the foundation for frequency-domain characterization and resonance frequency identification of the plant’s dynamic response. It also forms the foundation for future investigations aimed at active vibration control and resonance avoidance during drilling operations.

Reliable dynamic simulation of ACME requires precise identification of its physical parameters, as these values directly affect model accuracy and the validity of subsequent analyses. The essential parameters encompass the lumped mass of each link, the positions of their centers of mass (CoM), principal moments of inertia, and the stiffness and damping properties of the compliant joints. The mass, CoM, and Moment of Inertia parameters were obtained from the comprehensive CAD model that was created during the prototyping stage. The mass and geometric data of each component were used to calculate the assembly’s total mass considering material density and volume, while CoM locations were determined according to the spatial distribution of mass within each link. The extracted quantities, summarized in Table 4, form the primary input dataset for the MATLAB R2023b Simulink dynamic model, ensuring a realistic representation of ACME’s physical behavior.

**Figure 10:**
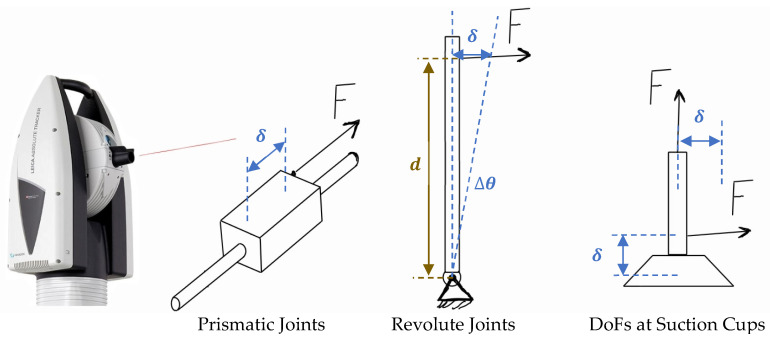
Experimental determination of joint stiffness through application of calibrated loads and precise displacement measurement using a laser tracking system.

The stiffness of each joint is experimentally determined by applying a series of controlled loads and measuring the resulting motion with high precision. As shown in Figure 10, incremental forces or torques are applied along the joint’s principal direction of movement, while the corresponding linear or angular deflections are recorded using a Leica laser tracker. The collected load–displacement data serve as the basis for accurately quantifying the stiffness of each joint.

After collecting the experimental data, the stiffness parameters are extracted through linear regression of the measured load–deflection responses. For prismatic joints, the stiffness is determined from the slope of the applied force versus displacement curve, while for revolute joints, it is derived from the torque versus angular deflection relationship. The stiffness of a linear joint is calculated as follows:

(27) $$K_{i}=\frac{F_{i}}{\delta_{i}}$$

(28) $$K_{r}=\frac{\tau }{\theta }$$

In these formulations, $F_{i}$ represents the externally applied linear load (in newtons), while $\delta_{i}$ corresponds to the measured linear deformation (in meters). Similarly, the rotational stiffness of each revolute joint is evaluated by taking the ratio between the applied torque τ (N·m) and the resulting angular displacement θ (radians). The complete set of experimentally identified stiffness and damping values for all joints is provided in Table 5.

At the early stage of dynamic modeling, damping coefficients were approximated using engineering reasoning and prior experience with comparable systems. Although damping influences vibration amplitudes, stiffness values have a more significant impact because they define the system’s natural frequencies. Consequently, inaccuracies in damping coefficient estimation mainly affect the intensity of resonance peaks rather than the resonance frequencies themselves. Therefore, an informed initial assumption for damping is generally sufficient for preliminary simulations, with subsequent refinement achieved by iteratively comparing numerical predictions against experimental test data if needed.

**Figure 11:**
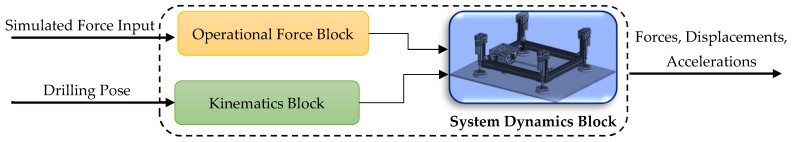
Schematic of the Simulink model.

The ACME’s dynamic model consists of three integrated subsystems, as shown in Figure 11.

- Kinematics Block: Computes the required joint configurations to achieve the desired tool-center-point (TCP) trajectories, ensuring precise motion during operation. This block also evaluates the kinematic matrices J (q), Coriolis/centrifugal matrix $C(q,\dot{q})$, and g(q).
- System Dynamics Block: Represents the physical characteristics and coupled behavior of the mechanism, including link masses, inertias, joint stiffness, and damping effects. It implements Equations (22)–(26), integrating motor-link coupling, joint stiffness, damping, and suction-cup compliance.
- Operational Force Block: Applies realistic external loads to the drilling nose by injecting either measured or commanded thrust and torque profiles, $w_{\mathrm{drill}}(t)$, into the model to reproduce actual operating conditions.

The resulting coupled model provides a comprehensive and computationally efficient framework for a kinematic–dynamic description of the system for time- and frequency-domain analyses of ACME’s dynamic behavior, pose generation, and a compliant multi-DoF plant realized in Simulink with experimentally identified parameters.

**Figure 12:**
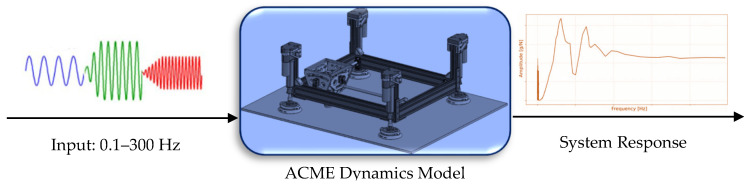
Schematic of the simulated FRF.

**Verification.** Prior to any employment of the developed numerical model for actuation, vibration analysis, or controller design, it is essential to confirm that the simulated dynamics accurately represent the physical behavior of the real system. This verification is carried out through Frequency Response Function (FRF) analysis, a well-established method for characterizing the dynamic properties of mechanical structures. In the numerical study, sinusoidal excitation forces were applied at the tool center point (TCP), that is, at the tip of the drilling nose, where drilling loads act in practice. Each excitation was applied along the corresponding Cartesian direction (X, Y, or Z), one axis at a time, to isolate directional modes. A total of 100 excitation frequencies, logarithmically spaced within the range of 0.1–300 Hz, were simulated in MATLAB Simulink. This range was selected to encompass the expected resonance regions of ACME, as the structure contains relatively large and high-inertia components, leading to dominant vibration modes in the low-frequency range. For each frequency and axis, the steady-state acceleration response was recorded at a virtual sensor node located 10 cm above the TCP, corresponding to the physical location of the triaxial accelerometer used in the experimental tests. The FRF magnitude was then computed as the ratio of acceleration amplitude (in g) to the input excitation amplitude (in N), producing an amplitude-versus-frequency plot for each direction. These simulated FRFs (see Figure 12) enable the identification of resonance frequencies and damping characteristics, with peaks corresponding to natural vibration modes governed by the combined stiffness and mass distribution of the system. A close correspondence between these simulated resonances and the experimentally observed ones validates the accuracy of the dynamic model and the fidelity of the identified physical parameters.

**Figure 13:**
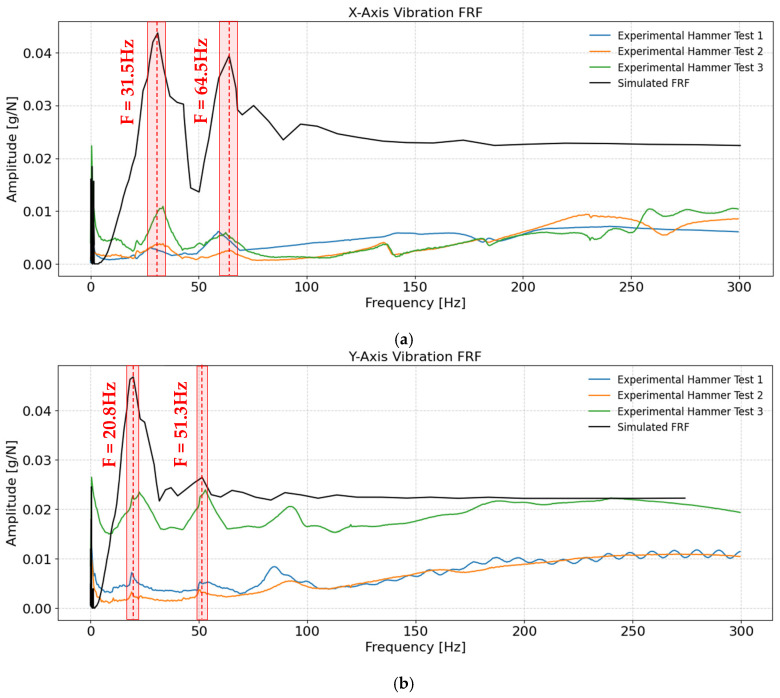

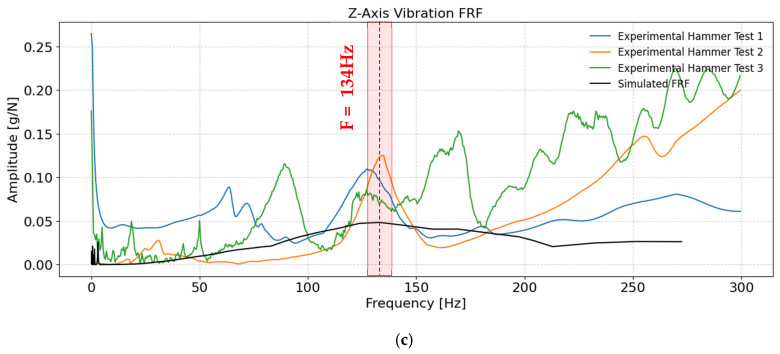
Experimental and simulated FRF (**a**) along X axis, (**b**) along Y axis, (**c**) along Z axis.

The frequency response of the ACME was characterized through both simulation and experimental impact testing, with results shown in Figure 13a–c for the X, Y, and Z axes, respectively. The simulated FRF was obtained from the dynamic model, while the experimental FRFs were measured using instrumented hammer tests with a triaxial accelerometer and multi-channel data acquisition. For the experiments, three independent hammer impacts were applied along each of the principal directions (X, Y, Z), ensuring that each excitation aligned with the corresponding axis of the end-effector. A mode was considered valid only if the associated resonance peak appeared consistently in all three repetitions for that direction. Comparison between simulation and experiment demonstrates the model’s capability to capture the dominant resonance frequencies observed in practice.

In the X-direction, dominant resonance peaks were observed around 31.5 Hz and 64.5 Hz in the simulation results, with the experimental data confirming these features. The agreement between the simulated and experimental modal frequencies was quantified using the root mean square error (RMSE), defined as the square root of the average of the squared differences between the simulated and experimental frequencies. To provide a scale-independent metric, the RMSE was normalized by the mean of the experimental frequencies and expressed as a percentage. For the first mode, the simulated frequency (31.5 Hz) compared with the three experimental values (27.6, 33.0, and 33.5 Hz) yields an RMSE of about 2.7 Hz and a normalized error of 8%. For the second mode (64.5 Hz simulated; 58.5, 62.1, and 64.0 Hz experimental), the RMSE is approximately 2.6 Hz, corresponding to a normalized error of 4.2%. The area around each mode corresponding to its normalized error distance is highlighted in red in Figure 13. While the modal frequencies match relatively well, a noticeable difference was observed between the amplitudes of the simulated and experimental frequency response functions (FRFs). This discrepancy primarily arises from the damping assumptions used in the numerical model. In the simulation, the damping coefficients were not identified or tuned, effectively representing a lightly damped condition. Consequently, the simulated FRF exhibits sharper and higher amplitude peaks than those measured experimentally. The lower amplitudes in the experimental FRF indicate that the real system experiences higher damping along this axis, dissipating more vibrational energy and reducing resonance magnitudes. Incorporating experimentally inferred damping characteristics in future models is expected to further improve the quantitative agreement between simulation and experiment.

For the Y-axis, the simulated and experimental modal frequencies also showed strong agreement. The first mode, with a simulated frequency of 20.8 Hz compared to the experimental values of 19.1, 19.0, and 22.5 Hz, yielded an RMSE of approximately 1.8 Hz (normalized error ≈ 8%). The second mode, at 51.3 Hz in simulation and 50.3, 50.8, and 53.3 Hz experimentally, produced an RMSE of 1.3 Hz (normalized error ≈ 2.5%). Similarly to the X-axis, the experimental FRF showed lower amplitude peaks, again implying higher actual damping than the undamped assumption of the model.

For the Z-axis, the simulated natural frequency (134 Hz) showed close agreement with the experimental results (136.6, 127.1, and 134.8 Hz), giving an RMSE of 4.8 Hz and a normalized error of 3.6%. The higher peak magnitude along this axis confirms that the structure is stiffer in the Z direction, consistent with its vertical load-bearing role. Two of the experimental FRFs, Test 2 and 3, display an increase in their amplitude in frequencies above 150 Hz, which does not occur in Test 1. This observation indicates reduced input–output coherence, which can be attributed to a reduced signal-to-noise ratio at higher frequencies, where the impact excitation provides limited energy and the structural response amplitudes are correspondingly small. This is likely due to the increased difficulty of achieving consistent hammer impacts along this axis, particularly when multiple or uneven impacts occur. Other factors influencing coherence are measurement noise, sensor mounting inconsistencies, and high-frequency artefacts introduced by the experimental setup. In this case, the response above 150 Hz is ignored since it is not considered representative of the true structural dynamics. Nevertheless, in all cases, the lower-frequency modes remain the most critical, as resonances near the drilling units’ operational speed (≈ 83 Hz) are of primary concern for ensuring vibration stability and minimizing dynamic amplification during drilling operations. The same trend of lower experimental amplitudes compared to the simulated FRF is present here, though slightly less pronounced than on the X and Y axes, again suggesting greater actual damping than the assumed simulation parameters.

Overall, the simulated FRFs successfully capture the primary resonances with good fidelity, while the experimental data reveal additional higher-frequency peaks attributable to secondary modes and local structural flexibilities not represented in the simplified model. Across all axes, the simulated peaks generally exhibit higher amplitudes than the experimental ones (except slightly along the Z-axis), as quantified in Table 6, primarily due to the initial estimation of damping parameters. Since damping predominantly affects vibration amplitude rather than resonant frequency, the higher simulated peaks further confirm that the true damping in the physical system is greater than the nominal values assumed in the model. For the purpose of this study, however, the accurate prediction of the natural frequencies is sufficient for resonance avoidance and dynamic performance assessment in low frequencies near the operating frequency of the Drilling Unit, while the detailed identification and tuning of damping parameters will form the scope of future research focused on system identification and parameter estimation, which lies beyond the objectives of the present work.

## 4. Cyber–Physical System with Digital Twins

**Figure 14:**
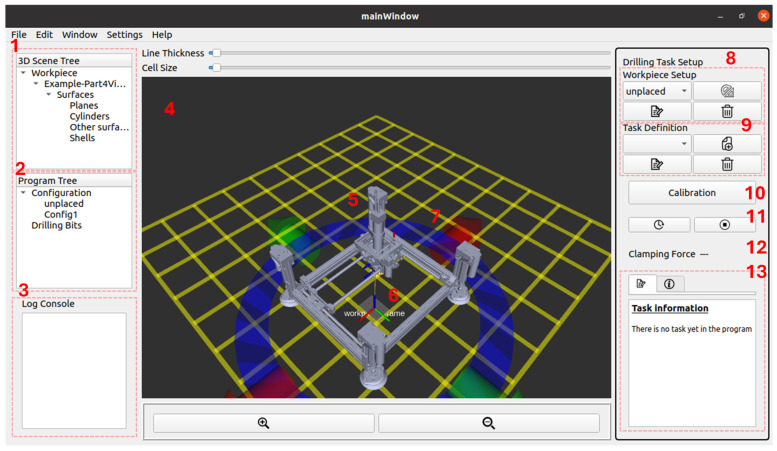
GUI layout for the developed CPS: 1: 3D scene tree of the loaded workpiece and robot, 2: Program tree for the tasks sequence, 3: Log console, 4: Render panel, 5: ACME DT, 6: Workpiece DT, 7: interactive markers, 8: Workpiece setup widgets, 9: Task definition widgets, 10: Calibration widget, 11: Task execute and stop buttons, 12: clamping force monitor, 13: Task information.

The drilling operation of ACME is executed and supervised through a cyber–physical system (CPS) framework that embeds digital twin (DT) representations of both the robot and the workpiece. This integration enables synchronization between the virtual and physical environments, supporting real-time task planning, monitoring, and verification. CPS architecture transforms ACME from a stand-alone end-effector into an intelligent, self-aware manufacturing tool capable of adaptive decision-making and operator-assisted control. A custom graphical user interface (GUI) has been developed to serve as the operator’s main interaction point with the CPS. As illustrated in Figure 14, the GUI incorporates a three-dimensional visualization of the digital twin environment, providing an intuitive workspace for defining and executing drilling operations. The interface includes several key panels:

- 3D Scene Tree (1): Displays all loaded entities, including the workpiece geometry, coordinate frames, and ACME DT.
- Program Tree (2): Organizes task sequences, configurations, and tool definitions.
- Log Console (3): Continuously records system messages, allowing the operator to monitor runtime data and diagnostic information.
- Render Panel (4): Visualizes the 3D digital twin scene in real time, showing both ACME and the workpiece.
- ACME DT (5) and Workpiece DT (6): Digital replicas that mirror their physical counterparts through ROS data streams.
- Interactive Markers (7): Enable manual manipulation of the workpiece and tool poses in 3D space. For example, the blue ring in Figure 14 allows rotation of the workpiece about the body-frame $Z_{w}$ axis, while the red and green arrows enable translation along the $X_{w}$ and $Y_{w}\;$ axes, respectively.
- Setup and Control Widgets (8–13): Manage workpiece setup, calibration, drilling task definition, clamping-force monitoring, and execution feedback.

This CPS was implemented using the Robot Operating System (ROS Noetic) for modular communication and data exchange, combined with Qt for GUI design. Visualization is built upon RViz, chosen for its lightweight rendering performance compared to more resource-intensive platforms such as Unity3D or Isaac Sim, an essential feature for deployment on embedded processors like the Raspberry Pi 4.

**Figure 15:**
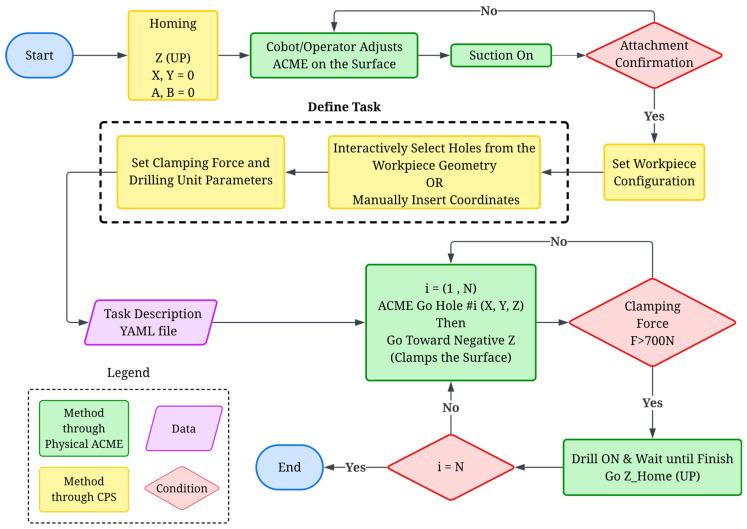
Operating flowchart.In system calibration and homing phase, all prismatic and tilting joints of the ACME (X, Y, Z, A, B) are driven to their reference home positions (0, 0, 0, 0, 0) to establish the origin of motion. The home state corresponds to the lower-left corner of the X–Y carriage range, the uppermost point of the Z-axis linear actuators, and the mid-rotation point for the A–B tilt axes, which are designed symmetrically about their range limits. For the X–Y CoreXY mechanism, the Dynamixel motors operate in constant-velocity mode, and the Average Torque Feedback (ATF) is monitored as a proxy for contact detection. As illustrated in Figure 16a, Step 1 drives the X carriage leftward until the ATF reaches 50% of its nominal torque (default value), indicating contact with the mechanical hard stop and halting motion. The 50 Hz feedback rate and low velocity ensure prompt and precise stopping. The ATF threshold and velocity can be adjusted by the operator as needed. In Step 2 (Figure 16a), the Y carriage undergoes an identical motion toward the lower end of the frame while maintaining the X position, thus defining the home position for the X–Y plane. The Z-axis homing sequence raises the linear actuators to their upper limit, detected by a limit switch, ensuring a safe clearance between the drilling nose and the surface while maintaining the 7 cm sensor-to-surface distance, the optimal spacing for high-quality imaging and point-cloud capture. For the A–B tilt axes, both joints move at constant velocity toward their lower and upper mechanical stops, with torque feedback monitored in a manner similar to the X–Y calibration routine. Upon reaching each limit, the system records the range of motion, and the midpoint of each range is assigned as the home position for the respective axis (see Figure 16b).

The workflow for conducting a drilling operation through the CPS is outlined in Figure 15. It details the complete sequence, from system initialization to task completion, and highlights how physical actions (green), cyber-level processes (yellow), and data management (purple) interact under defined logical conditions (red diamonds). Upon completion of the homing stage, the operator positions ACME onto the work surface using the cobot arm manually or via robot programming. Once in place, suction activation is commanded to secure the device to the surface, and an attachment confirmation signal validates proper sealing before proceeding. The end-user may set the configuration of the workpiece manually or via interactive markers. In the task definition phase, the operator sets the clamping force and drilling parameters, such as feed rate and spindle speed, through the GUI. The system allows two modes of defining drilling positions: (i) interactive hole selection directly from the workpiece digital twin using markers (which serve as user interaction tools and are not part of the vision system), or (ii) manual coordinate input. Once the task parameters are finalized, they are compiled into a configuration file, ensuring repeatability and offline task reloading. During operation, the CPS iterates over each hole position (i=1 to N), where ACME moves sequentially to each defined coordinate. Upon reaching the target, the Z-axis actuators generate the clamping force, verified by the integrated load sensor (e.g., F threshold greater than 700 N). If the condition is satisfied, the drilling unit is activated, executing the programmed drilling cycle before retracting to its home position. The loop continues until all holes are completed. This logic encapsulates both decision-making and safety interlocks, ensuring that drilling operations cannot proceed without proper suction, surface attachment, and verified clamping. The CPS thus enables a structured, semi-autonomous drilling process where the operator’s role is limited to supervisory control rather than low-level command execution. More details regarding the operation stages are elaborated in the rest of this section.

**Figure 16:**
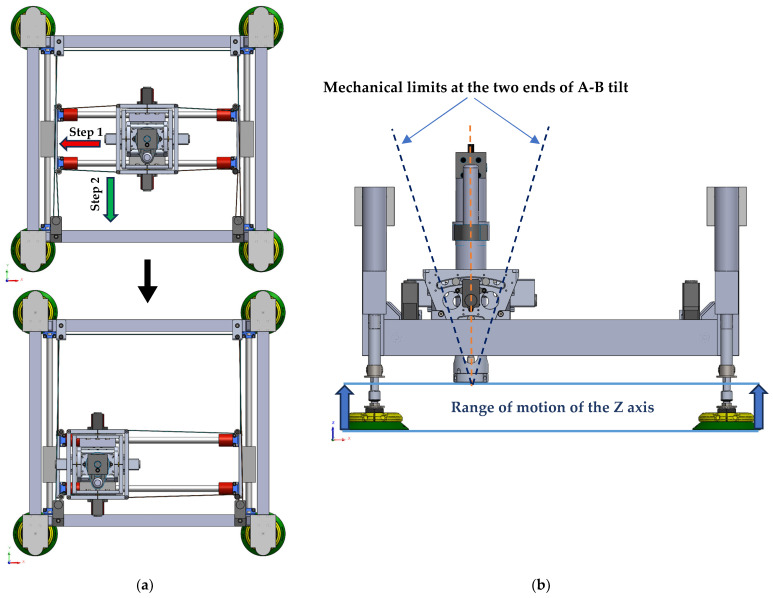
Calibration (homing) process for (**a**) X-Y axes in two steps, (**b**) for Z, A, and B axes.

**Figure 17:**
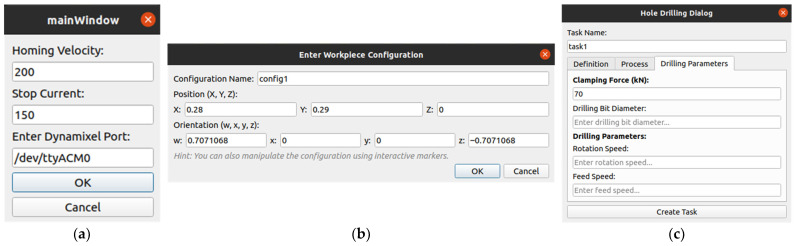
(**a**) Calibration settings dialog (**b**) Workpiece configuration dialog (**c**) Hole drilling dialog.

To facilitate user interaction, CPS provides dedicated configuration dialogs for calibration, workpiece configuration, and drilling parameters (Figure 17):

- Figure 17a shows the Calibration Dialog, which allows homing velocity, stop current, and communication port configuration for motor drivers. This ensures all joint encoders are zeroed properly before task execution.
- Figure 17b illustrates the Workpiece Configuration Dialog, where the operator defines or adjusts the workpiece pose, either manually through position and orientation fields or interactively through on-screen markers.
- Figure 17c presents the Hole Drilling Dialog, where drilling parameters such as clamping force, feed rate, and spindle speed are specified.

**Figure 18:**
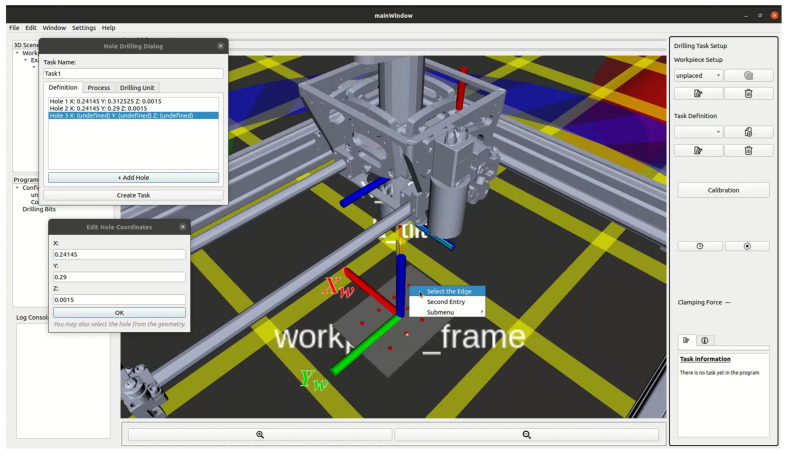
Interactive hole selection.

The CPS enables the operator to intuitively and interactively define the multi-hole drilling task within the 3D visualization environment. As shown in Figure 18, holes can be selected directly from the rendered digital twin of the workpiece, enabling the operator to plan the drilling sequence visually rather than by entering numerical coordinates. This functionality is powered by the Open CASCADE geometric kernel, which provides access to the parametric features of the loaded CAD model. Through this integration, the precise spatial coordinates of holes are automatically extracted from the 3D geometry and represented as interactive cylindrical markers in the scene. Each marker can be selected, repositioned, or modified in real time, significantly reducing setup time and minimizing operator error compared to manual coordinate entry.

The CPS has been designed and developed for future add-ons. When the computer-vision algorithms are fully developed, validated, and available, pilot holes coordinates will be extracted using the vision system. With ACME attached firmly to the surface, the vision system will be brought to inspect some pilot holes, extracting the holes’ coordinates with respect to the defined ACME’s coordinate frame and thereby locally registering the workpiece surface. Known drilling patterns, defined in the CAD model and including those pilot holes, will be adjusted and transferred to the ACME frame. These new coordinates will be written into a file, allowing the user to import the patterns into the task definition rather than manually selecting holes from the geometry. Hence, the cobot does not contribute to the positioning errors that are measured in experimental tests, and all errors are local to the ACME frame.

Once the drilling parameters and hole coordinates are set, the CPS automatically generates a structured YAML task file that synchronizes with both the physical ACME and its digital twin. This allows real-time monitoring of the drilling process while maintaining a consistent digital record of the operation. The sequence and pattern of drilling operations depend on the order in which the holes are defined, either through interactive selection from the visualized workpiece geometry or by importing a file containing the hole coordinates. The drilling unit executes the holes sequentially according to this predefined pattern. Thermal accumulation may not be significant due to the short drilling duration relative to the transition time between holes and the normalization period. Nevertheless, to accommodate thermal dissipation or other operational considerations, the operator can select the order of drilling holes in the GUI according to a desired pattern. Alternatively, if the imported file specifies a drilling sequence optimized for thermal management, the CPS automatically executes that pattern.

**Figure 19:**
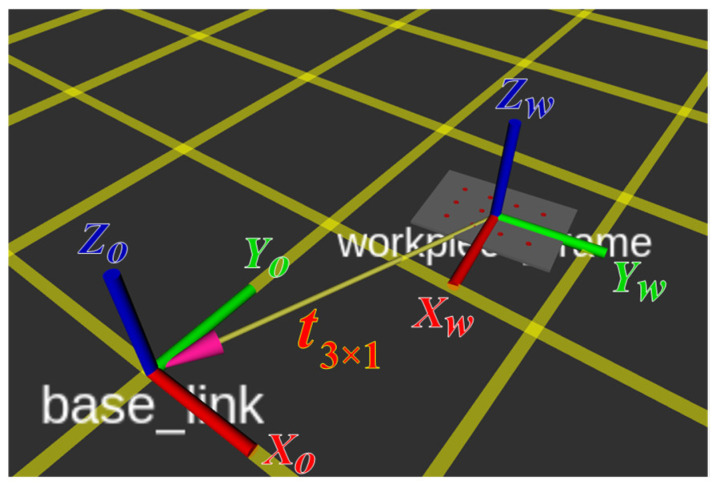
Workpiece position and orientation w.r.t. the base space-fixed frame.

Since the extracted hole positions are defined in the workpiece coordinate frame {W}, which may not coincide with the ACME base frame {0}, a transformation is required before visualization and execution. The relation between the two frames is illustrated in Figure 19, where the workpiece pose is defined by a translation vector $t_{3\times 1}$ and a rotation matrix $R_{3\times 3}$. The transformation of a point from the workpiece frame to the ACME base frame is given by:

(29) P01=TW0PW1, TW0=R3×3t3×101×31

Here, (TW0) represents the homogeneous transformation between the workpiece and the ACME base frames. The rotation matrix $R_{3\times 3}$ is derived from quaternion-based orientation data to ensure smooth, singularity-free representation and interpolation during interactive manipulation. Quaternions are particularly advantageous in this context because they eliminate gimbal-lock issues inherent to Euler angles and are natively supported by ROS TF and RViz frameworks. The corresponding rotation matrix expressed in terms of quaternions $q=[q_{w},q_{x},q_{y},q_{z}]$ is given by:

(30) $$R_{3\times 3}=\left[\begin{array}{ccc} 1-2q_{y}^{2}-2q_{z}^{2} & 2q_{x}q_{y}-2q_{z}q_{w} & 2q_{x}q_{z}+2q_{y}q_{w}\\ 2q_{x}q_{y}+2q_{z}q_{w} & 1-2q_{x}^{2}-2q_{z}^{2} & 2q_{y}q_{z}-2q_{x}q_{w}\\ 2q_{x}q_{z}-2q_{y}q_{w} & 2q_{y}q_{z}+2q_{x}q_{w} & 1-2q_{x}^{2}-2q_{y}^{2} \end{array}\right]$$

**Figure 20:**
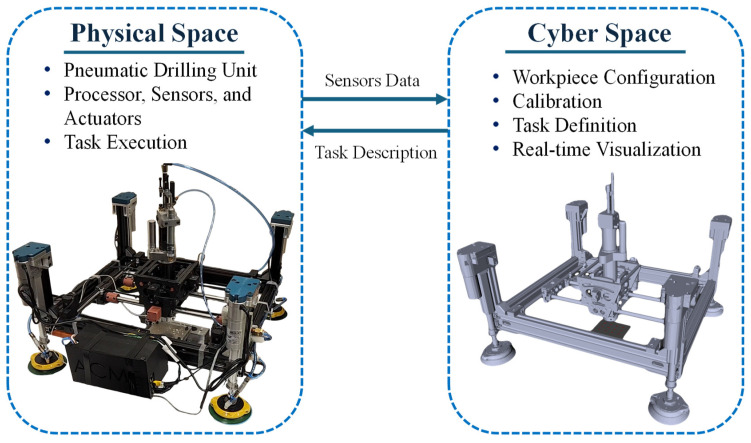
Physical space and cyber space communications and roles. The user defines the task in the CPS and sends it to the ACME, while encoders send data to the DT to update joints.

Cyber space and physical space operate in continuous, bidirectional communication, forming a tightly coupled loop that enables real-time synchronization between ACME and its digital twin (DT). As illustrated in Figure 20, the operator interacts within the cyber environment, defining the drilling task, setting process parameters, and commanding execution, while the physical ACME responds through coordinated actuator motions. Simultaneously, sensor and encoder feedback from the physical hardware are streamed back to the CPS, updating the DT’s joint states in real time. This ensures that the virtual model mirrors the actual pose and motion of the end-effector at all times, maintaining geometric and temporal coherence between both domains.

To achieve efficient rendering on embedded platforms such as the Raspberry Pi 4, the polygonal complexity of the ACME 3D model was reduced using MeshLab. This lightweight representation allows the system to sustain continuous visualization updates without sacrificing responsiveness or synchronization accuracy. The DT therefore serves not only as a visualization aid but also as a diagnostic and monitoring interface, displaying live actuator motion, clamping status, and processing feedback directly within the GUI.

**Figure 21:**
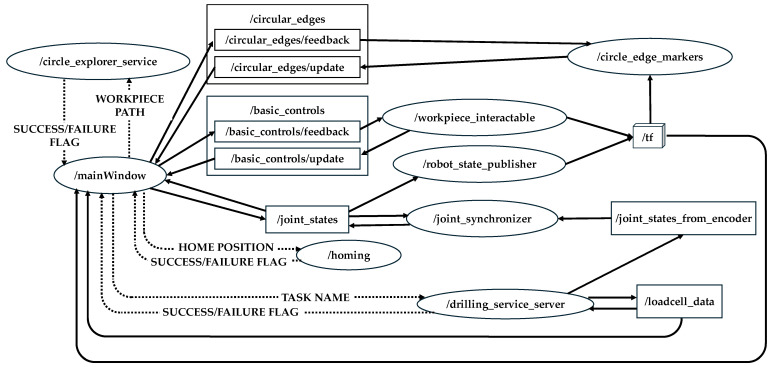
Communications between ROS nodes and topics, based on rqt_graph. Solid arrows and dashed arrows represent publisher/subscriber and service/client relationships respectively.

The underlying communication between the physical and cyber layers is orchestrated through the Robot Operating System (ROS) framework, which handles message passing, node management, and service calls. The network of ROS nodes and topics used within the CPS is depicted in Figure 21, generated using the rqt_graph tool. This architecture separates GUI-level interactions from low-level actuation and sensing, ensuring modularity, scalability, and maintainability.

A detailed summary of the implemented communication structure is presented in Table 7, which maps each user interface feature to its corresponding ROS services (srv), clients (clnt), publishers (pub), and subscribers (sub). For example, the workpiece model upload feature relies on the/circle_explorer_service to extract hole coordinates from STEP/STL geometry files, while interactive drilling-hole selection is enabled by the/circle_edge_markers node that generates cylindrical markers at detected hole locations. Similarly, joint synchronization between the physical system and the DT is achieved through the/joint_synchronizer node, which subscribes to encoder feedback published to/joint_states_from_encoder and updates the rendered ACME model accordingly. Another key feature is the clamping force monitor, which utilizes a load-cell feedback loop: the/drilling_service_server reads the force data from the sensor, publishes it on the/loadcell_data topic, and subscribes to the same topic to manage automated clamping termination when the threshold is reached. The same topic is also visualized in the GUI for live force monitoring by the operator.

The modular development of the CPS enables seamless customization of the platform for diverse manufacturing tasks and different robot models. To adapt it for other applications such as finishing, riveting, and inspection, prospective developers only need to modify a few GUI widgets, such as the task definition dialog, and add or remove a limited number of ROS topics. Most core features, including workpiece configuration, joint synchronization between the physical system and its DTs, render panel interaction, task description widget, and log console, remain unchanged. For instance, one intends to develop a CPS for finishing operations, may reuse the existing implementation and simply integrate a planning module to generate an optimized tool path. For riveting, the operator may select the corresponding hole locations on the visualized workpieces to be joined, in the same interactive manner used for selecting holes from the geometry during the drilling operation. Furthermore, any other robot model or end-effector can be incorporated into the system by replacing the Unified Robot Description Format (URDF) file or including a Semantic Robot Description Format (SRDF) file to enable path planning. The CPS also supports importing arbitrary workpieces through STL or STEP files and interacting with them regardless of the application.

Collectively, this architecture enables closed-loop coordination between the real and virtual systems, where commanding, execution, sensing, and visualization occur concurrently and transparently to the operator. The modular node–topic structure further allows straightforward integration of additional sensors, controllers, or analytical modules, laying the groundwork for future extensions such as predictive maintenance, anomaly detection, and adaptive vibration suppression through the digital twin framework.

## 5. Experimental Drilling Results

More than 300 holes were drilled using both ACME alone and with ACME mounted on a UR10e cobot. All the drilling tasks were defined and executed through the CPS operated by multiple users and technicians with no programming knowledge, and none of them reported any discomfort using the developed system. To evaluate the real-time performance of the DT-enabled CPS, the average latency, sensing and control update rates, visualization frequency, and computational load were measured. The system exhibited an average communication latency of 76 ms between the physical system and its digital twin. The motor encoders operated internally at 120 Hz, providing high-fidelity position feedback for motion monitoring, while the loadcell readings, acquired through the HX711 analog-to-digital converter (ADC) module, were sampled at 80 Hz for real-time clamping-force estimation. The DT visualization layer refreshed at 20 Hz, ensuring smooth and responsive rendering of the physical system’s state. Hence, The DT-enabled CPS maintains an overall refresh rate of 20 Hz, mainly limited by visualization performance, while its internal sensing and control processes run at higher update rates to ensure high-quality drilling. Resource profiling on Raspberry Pi 4 (8 GB RAM) indicated an average CPU utilization of 68% and memory usage of 41% during full operation. The polygonal simplification of the ACME 3D model in MeshLab further enhanced rendering efficiency, enabling near-real-time visualization on embedded hardware while preserving spatiotemporal synchronization with the physical system. To quantitatively investigate ACME’s performance in positioning and normality accuracy, this section discusses the results of one of the drilled stacks in depth.

A total of 25 holes were drilled on a 3-layered coupon composed of 2 mm aluminum sheets. The air stream was turned on so that the suction cups would engage. Within the CPS GUI, the operator chooses the CAD model of the coupon in the prompt window, which was then rendered in the visualization panel for selecting its geometric elements. After setting the position and orientation of the workpiece in the CPS, the user interactively selected the holes to be drilled from the coupon model and defined a multiple-hole drilling task. Moreover, the clamping force of 1000 N was set for this test. As the drilling task was initiated via CPS, the carriage moved to the first hole’s X and Y coordinates, and then the linear actuators lowered the drilling unit to the coupon surface. Since the suction cups were activated, the clamping force started to build up, and once the threshold of 1000 N was reached, the drilling began for the first hole. Upon drilling the hole, the linear actuators retracted the drilling unit to its initial Z position, and then the carriage proceeded to the next hole. The same procedure was repeated for all 25 holes.

The drilling operation was performed using a 0.50 HP Ingersoll Rand pneumatic auto-feed drilling unit (DU) specifically designed for aerospace applications. The DU was supplied with compressed air at 90 psi and operated at a nominal spindle speed of 5000 RPM, corresponding to a rotational frequency of 83 Hz. The unit was equipped with a 5 mm-diameter uxcell Jobber Twist Drill Bit made of black nitride- and titanium-coated 4341 HSS, featuring a 135° split-point geometry suitable for aluminum drilling. The feed rate of the auto-feed spindle was set according to the manufacturer’s nominal feed range for this speed (theoretical value), providing a constant feed motion during each drilling cycle. At this spindle speed, the fundamental excitation frequency associated with the cutting process is approximately 83 Hz, with its second harmonic near 166 Hz. Using the validated dynamic model presented in Section 4, these frequencies were verified to be well separated from the system’s resonance frequencies, which occur mainly below 70 Hz in the X–Y plane and around 130–140 Hz along Z. Consequently, the drilling excitation does not coincide with the dominant resonant modes of ACME, ensuring stable operation and avoiding dynamic amplification during drilling.

It should be noted that the objective of the drilling test was to evaluate whether ACME can maintain stable performance without focusing on achieving a certified level of hole quality. The drill bit was selected from commercially available general-purpose bits without any specific design. The drilling parameters were selected as an initial guess based on the available parameters of the drilling unit. Generally, in aerospace automated drilling, none of these parameters are overlooked; the drill bit is custom-designed for a specific range of rotational speeds and material stack-ups; the drilling unit is purchased to satisfy the required specifications; process enhancements such as lubrication and vibration-assisted drilling technologies are considered; and after conducting characterization tests, the final drilling parameters are refined and precisely specified. Achieving this level of refinement requires significant effort and lies beyond the scope of this paper. Such optimization would need to be performed in collaboration with aerospace industry partners to meet application-specific requirements.

**Figure 22:**
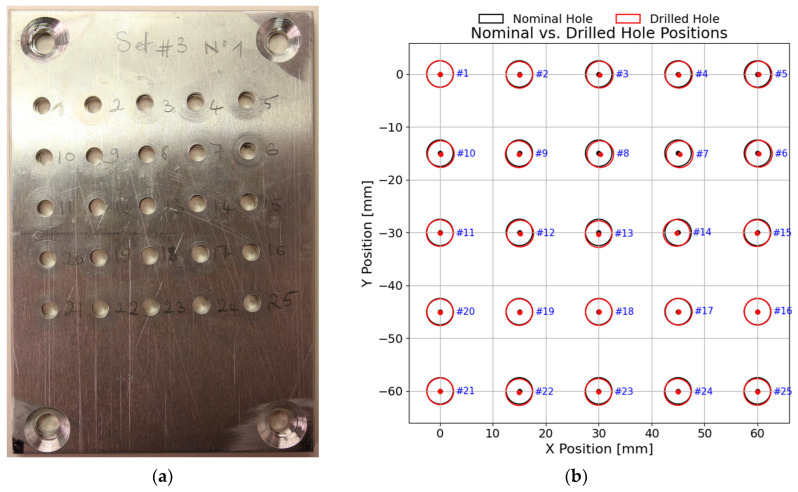
(**a**) drilled holes on the three-layer metallic stacks with their indices (**b**) visual comparison between the drilled holes and the nominal holes.

Figure 22a demonstrates the stacked aluminum sheets after all the holes were drilled. The hole indices imply the sequence of the carriage movement, and they are also used for analysis in this section. The four corners of the workpiece were manually drilled and countersunk to keep the layers attached while preventing collisions between the drilling unit’s nose and the protruding fastening bolts in these areas. The quality of the drilled holes was evaluated using a Mitutoyo MACH 806 Coordinate Measuring Machine (CMM), and the hole diameter, cylindricity, as well as errors in positioning and normality for all holes were gathered. Figure 22b presents a visual comparison between the drilled holes and the nominal holes, showing a close correspondence.

**Figure 23:**
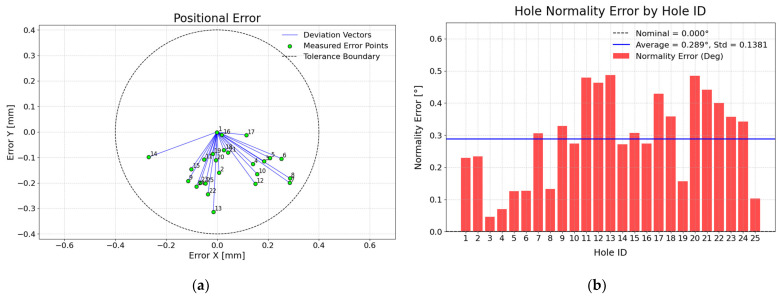

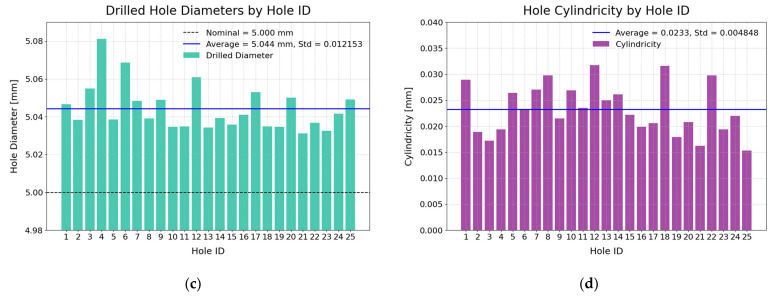
(**a**) Positioning error in X and Y coordinates of each hole with the hole ID at the top-right corner (**b**) Normality error for each hole and the average value (**c**) Drilled hole diameters for each hole, along with the average value (**d**) hole cylindricity for each hole, along with the average value.

Figure 23 provides an overview of the geometric quality results obtained from the drilling experiments. The plots illustrate the measured positioning deviations, angular (normality) errors, final hole diameters, and cylindricity values for all drilled holes in the test sample. This visualization offers an intuitive representation of the distribution and consistency of the results across the 25 holes. While individual metrics such as positioning, angular accuracy, and hole geometry remain within typical aerospace tolerances, the detailed statistical evaluation of these parameters is presented in the subsequent process capability analysis. To estimate the measurement error bars, a conservative bounding uncertainty was calculated using the CMM’s accuracy specified in the datasheet,

(31) E=(3.5+4L/1000) μm

where L is the measuring span in millimeters. For the sample test sheets with a 5×5 grid, the maximum measurement span along each axis was approximately L=60 mm, which, when substituted into the accuracy expression, leads to an uncertainty of E=3.74 μm. This ±E bound is orders of magnitude smaller than both the measured displacements and the tolerance thresholds considered in this study, and therefore does not materially influence the interpretation of the results.

Positioning deviation quantifies how far the drilled hole center is from its nominal target location, providing a direct measure of translational accuracy. Angular or normality error assesses the misalignment between the drill axis and the intended surface normal, capturing the angular precision of the drilling approach. The final hole diameter is used here to characterize the dimensional accuracy of the drilled features compared to the nominal 5 mm tool, reflecting both tool performance and structural rigidity during cutting. Cylindricity evaluates how closely the full 3D surface of each hole conforms to a perfect cylinder by accounting for roundness, straightness, and taper along the hole’s depth; lower values indicate better geometric form. Together, these metrics provide a comprehensive assessment of positional, angular, dimensional, and geometric performance of the drilling process.

To rigorously evaluate the system’s geometric accuracy and stability, a statistical process performance analysis was performed on the key quality indicators extracted from the drilling experiments. For each metric, the process mean (μ) and standard deviation (σ) were computed (Table 8), and the upper and lower specification limits (USL/LSL) were defined according to the required geometric tolerances. The specification limits represent the allowable range within which a measurement is considered conforming.

Based on these limits, the process capability index (Cpk) was calculated. Cpk quantifies how well the process performs relative to the specification window by incorporating both the process variability and the degree to which the measurements are centered within the allowable range. In our case, Cpk provides the assessment of the ACME’s ability to consistently meet geometric and dimensional tolerances under the tested operating conditions. Although ±2σ control limits were used in the plots to improve visual clarity at the prototype stage, all Cpk values were computed using the full standard deviation and the actual specification limits.

**Figure 24:**
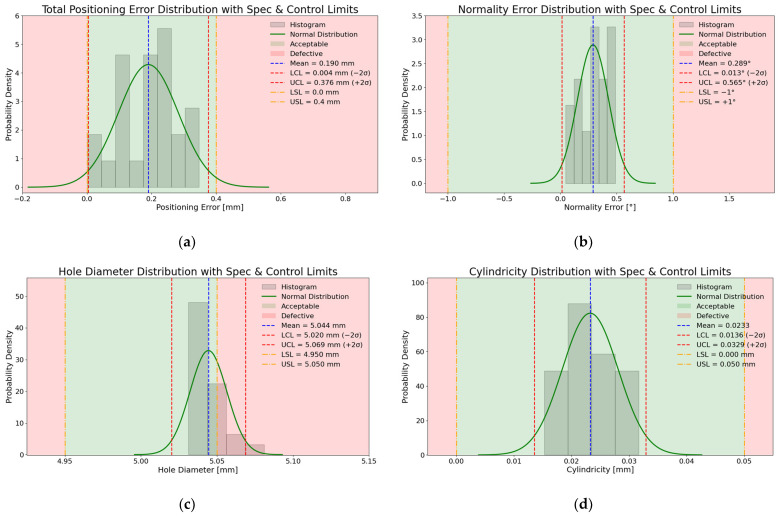
Statistical distribution of geometric and positioning errors of drilled holes, including control/specification limits of (**a**) total positioning error and (**b**) normality error (**c**) hole diameter and (**d**) cylindricity.

For the total positioning error, the system achieved a Cpk of 0.681 (Figure 24a), indicating limited positional capability and a relatively high number of defects per million opportunities (DPMO). This outcome is consistent with the prototype configuration, where positioning relied solely on the internal motor controllers and encoders, without external linear feedback. Despite this limitation, the prototype still satisfied the defined upper specification limit of 0.4 mm. Incorporating external linear encoders and a unified closed-loop controller that integrates all sensor feedback would significantly enhance positional accuracy in future iterations. The normality error and cylindricity metrics achieved Cpk values of 1.716 and 1.599, respectively, demonstrating reliable angular alignment within the ±1° prototype requirement and strong conformity to the cylindricity tolerance. In contrast, the hole diameter exhibited a low Cpk of 0.154, indicating that the selected drill bit size was not well matched to the specified tolerance range. However, Figure 24c shows that the diameter distribution is tight (σ = 0.012153 mm); selecting a slightly smaller drill bit (e.g., 4.956 mm) would shift the mean to approximately 5.0004 mm and raise the Cpk to 1.361. This demonstrates that the system is inherently capable of producing high-quality hole diameters when the tooling is properly selected.

Collectively, these results show that while several metrics already meet or exceed tolerance requirements, others can achieve acceptable capability with minor hardware or parameter adjustments. In precision manufacturing, Cpk values in the range of 1.3–1.5 and above are commonly regarded as indicative of a capable, production-ready process, corresponding to fewer than 3.4-63 DPMO.

**Figure 25:**
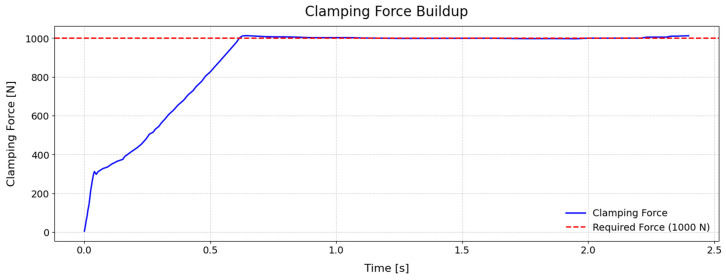
Exerted clamping force measured using a dynamometer.

To verify whether the required clamping force is generated using this novel design, the workpiece is configured with a dynamometer to measure the exerted force. Figure 25 shows that the clamping force begins to rise immediately when the drilling nose first contacts the surface (time = 0 s), reaching approximately 330 N by 0.05 s. This level has been sufficient in this test to initiate the tilting and passive self-alignment of the A–B mechanism from its ±10° limits toward the local surface normal (0° in this case). The linear actuators continue to increase the force until the clamping threshold of 1000 N (±5%) is achieved at around 0.62 s, keeping the total alignment time under one second. In practice, an additional 1–2 s is allocated for the system in the CPS to settle and ensure full stability before drilling begins. After this settling period, the system actively maintains the required clamping force as the drilling operation starts.

**Figure 26:**
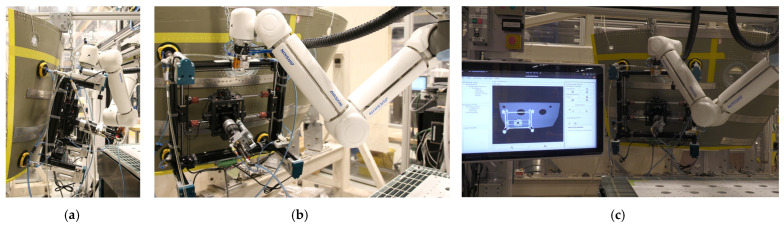
(**a**) Side view, (**b**) isometric view, and (**c**) operator desk view of the ACME mounted on a UR cobot drilling a fuselage section. Suction cups are fully attached to the double-curvature surface.

Figure 26 showcases ACME mounted on a UR cobot as an end-effector, which is positioned and attached to a simulated cockpit panel. This stacked UR–ACME robotic configuration enables safe collaboration with human operators while providing the required clamping force for multi-layered metallic double-curved surfaces via direct suction cup contact, a capability that the UR cobot alone cannot deliver. This is achieved without compromising the positioning or normality accuracy of the drilling process. In addition, by leveraging the cobot’s extended reach, ACME can reach multiple regions of the fuselage efficiently.

Table 9 provides a consolidated assessment of ACME’s performance relative to the established and the ideal threshold targets discussed in Table 1. The results show that ACME meets or surpasses most key requirements, including workspace size, setup time, clamping force, and cost, while remaining within acceptable limits for positioning and normality errors. Although the prototype achieves only marginal compliance with the ideal positioning tolerance, it still satisfies the practical requirement of 0.4 mm and demonstrates full compliance for the ±0.5° normality criterion. Overall, the table highlights ACME’s ability to deliver near-aerospace-grade performance at a fraction of the cost of conventional automated drilling systems.

**Figure 27:**
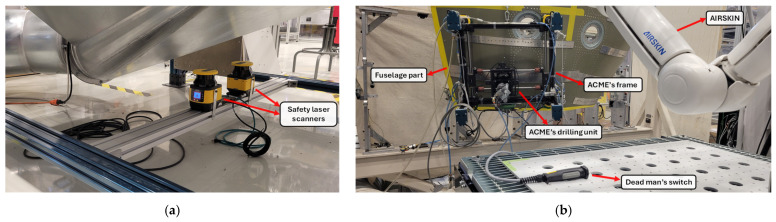
Safety interlocks: (**a**) safety laser scanners, (**b**) dead man’s switch and AIRSKIN protective layer for UR10e cobot.

As CPS is designed for automatic drilling operations in close proximity to human operators, safety measures play a critical role in preventing unintended motion or hazardous interactions. Multiple layers of protection are implemented at both software and hardware levels. Several thresholds are defined for key parameters such as force, speed, motor current, and drilling-unit inclination angle. Any deviation beyond these predefined limits triggers the CPS to enter a dedicated safety mode, which immediately stops ongoing motion and suppresses actuator commands as appropriate for each subsystem. Depending on the detected condition, certain actuators may be torque-disabled, while others may transition into back-drive or free-drive states to avoid rigid entrapment and allow safe manual repositioning. This ensures that, in the unlikely event of contact or obstruction, the system does not rigidly hold its position. Sensor validation routines continuously monitor incoming data from the load cell, encoders, and other feedback sensors. If the readings are found to be inconsistent or physically implausible, the system automatically transitions to the safety mode. Hardware interlocks shown in Figure 27 further enhance operational safety. A safety laser scanner continuously monitors the surrounding workspace and conditions the robot’s motion on both the presence detection and the state of the dead-man’s switch. When no operator is detected within the defined safety zone, the robot is permitted to operate autonomously without requiring the dead-man’s switch to be pressed. However, when a person enters the monitored area, robot motion becomes conditional on the dead-man’s switch being actively pressed by the operator. If the switch is released with the operator in the zone, the CPS immediately switches to its safety mode. In addition, if needed, the operator can instantly stop the system in emergency conditions. Furthermore, the UR10e collaborative robot is equipped with AIRSKIN, an active pressure-sensitive covering that immediately halts the system upon any unexpected physical contact, thereby ensuring a compliant and inherently safe collaborative environment.

## 6. Conclusions

This work introduced ACME, a lightweight, cobot-compatible end-of-arm drilling tool integrated within a cyber–physical system (CPS) and an interactable digital twin (DT) to enable jig-less aerospace drilling with high accuracy and operator accessibility. Mechanically, ACME combines vacuum-assisted clamping that can provide up to 1000 N of clamping force, a passive self-normalization nose for double-curvature alignment, and a compact 5-DoF architecture with CoreXY planar motion; electronically, it leverages embedded sensing and pneumatic control to synchronize force buildup with the drilling process. On the software side, the CPS/DT allows operators without programming expertise to import a workpiece model, interactively select hole locations, configure process parameters, and monitor execution in real time. Across more than 300 drilled holes—and in a representative 25-hole coupon test—the system achieved an average positional error of 0.1901 mm (maximum 0.3472 mm), an average normality error of 0.289° (maximum 0.487°), a mean hole diameter of 5.0444 mm, and an average cylindricity of 0.0233 mm, all within or close to typical aerospace tolerances, validating both the mechanical design and the CPS-guided workflow. The study also developed kinematic and dynamic models of ACME and verified their fidelity via simulated and experimental frequency-response analysis, capturing the dominant resonance behavior and informing parameter selection (e.g., joint stiffness/damping) for design and control. These results demonstrate that collaborative robots, when augmented with ACME and an operator-centric CPS/DT, can deliver aerospace-grade drilling performance while reducing footprint, cost, and commissioning complexity. An overview of the CPS and ACME drilling operation is demonstrated in Video S1.

In future work, the near-term goals will focus on (i) vision-based workpiece registration and automated alignment to minimize setup time to enable fully automated input generation (hole pattern creation); (ii) process enhancements, including integrated lubrication/coolant and the integration of Advanced Drilling Units (ADUs) with adaptive feed-rate and force control, to support broader material stacks (e.g., CFRP/Ti) and ensure high-quality drilling; and (iii) migration of the digital-twin (DT) environment to higher-fidelity yet lightweight simulation platforms (e.g., implementation utilizing NVIDIA Isaac Sim on an NVIDIA Jetson Series processor) and expansion of its functionality for quality analytics, automatic computation of heat-efficient drilling trajectories to minimize thermal effects, and enhanced safety interlocks.

These steps are critical to achieving a fully automated, multi-material-capable, and scalable platform suitable for industrial integration. The long-term goals will also address (iv) active vibration control with refined damping identification to enhance drilling quality and geometric fidelity and (v) extended validation on large, double-curvature fuselage sections under varying environmental and process conditions, along with long-duration reliability studies. These efforts will further mature ACME from a validated prototype to a modular, production-ready solution for reconfigurable aerospace manufacturing.

## 7. Patents

The patent PCT/IB2025/058659 was filed on 27 August 2025, as a result of this work, and it is pending as of the time this manuscript was submitted.

## Figures and Tables

**Table 1:** Quantitative values of the technical Specifications of ACME and other robotics solutions.

Characteristics	Objective	Threshold for ACME Prototype	Ideal Threshold for ACME	KUKA KR210	Crawling by MTorres [12,14]	Flex Track by Boeing [45]
Weight (M)	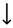	20 kg	M≤15 kg	A 100–200 kg EoAT [46]	50 kg	N/A
Setup Time ($T_{s}$)	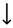	3 min	$T_{a}\leq 2\;min$	Very Long	~5 min	~10 min
Positioning time of the drilling head ($T_{p}$)	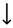	15 s	$T_{p}\leq 10\;s$	N/A	N/A	N/A
The Longest Workspace Dimension (LW)	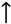	300 mm	LW≥300 mm	3100 mm	Varies by design	Varies by rail length
Minimum clamping force ($F_{c}$)	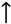	700 N	$F_{c}\geq 1000\;N$	2100 N [46]	1370 N	N/A
Positioning Error (P-*E*)	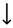	P-*E*: 0.4 mm	P-*E* ≤0.2 mm	±0.05 [46]	±0.2	±0.25
Normal Error (N-E)	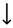	N-E: 1°	N-E ≤ 0.5°	<±0.5°	±0.5°	±0.5°
Cost (C) [CAD]	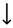	$25,000	C≤$25,000	>$2 million	N/A	N/A

**Table 2:** Summary of the main specifications of ACME.

Specification	Value	Description
Mass	~18 kg	Weight of the entire ACME (Including Drill Unit)
Overall Dimensions (L × W × H)	~655 × 684 × 588 mm	Approximate bounding box when Z axis is fully extended (home pose)
Maximum Workspace (L × W × H)	~302 × 237 × 76 mm	The range of motion of the X–Y–Z per axis
Optimal Workspace (L × W × H)	~267 × 212 × 76 mm	Recommended travel range for consistent performance
Maximum Useful Workspace (%)	~46% along X ~35% along Y 100% along Z	302/655 ≈ 46.1% along X and 237/684 ≈ 34.6% along Y, of the total device footprint is used as workspace
X–Y Axes Maximum Force	250 N	Force on the X–Y carriage system
X–Y Axes Maximum Speed	3.7 m/min	Translational speed of the CoreXY mechanism
A–B Axes Maximum Torque	58 N.m	Combined pitch (A) and yaw (B) axes torque around the drill axis
A–B Axes Maximum Speed	5.5 rev/min	Approximate rotation speed of the tilt axes
A–B Rotation Span	±10° each axis	Tilt range about the tool axis (home pose = 0°)
Z-Axis Typical Speed	~6–10 mm/s	Maximum possible velocity
Maximum Achievable Clamping Force	~up to 2360 N	Limited by the lifting force of the suction cups
Maximum Optimal Clamping Force	1000 N	Recommended operating clamping load to ensure longevity and safety
Electrical Supply	12 V DC @ 5 A	Power input for linear actuators, electronics, actuators, relays, display, etc.
Air Pressure Supply	Min. 90 psi (620 kPa)	Required for the drilling unit and vacuum generator
Communications Interfaces	USB/TTL, GPIO	Dynamixel TTL bus, Raspberry Pi GPIO for load cell & actuator relays, USB for Pi console

**Table 3:** 5-DoF ACME DH Parameters.

	J1 (Z-Axis)	J2 (Y-Axis)	J3 (X-Axis)	J4 (B-Axis)	J5 (A-Axis)
a [mm]	0	37	0	75.7	0
d [mm]	$d_{1}$ + 17.95	$d_{2}$ + 179.47	$d_{3}$ + 123.9	0	0
α [rad]	0	−pi/2	−pi/2	−pi/2	pi/2
θ [rad]	0	−pi/2	pi	$\theta_{4}$ (B) − pi/2	$\theta_{5}$(A) + pi/2

**Table 4:** ACME components’ mass and inertia parameters.

Link	Mass [kg]	CoM [m]	Moments of Inertia [kg.m^2^]
Main Body (Frame)	10.398	[0.2947, 0.2882, 0.0856]	[0.7296, 0.8512, 1.4088]
Y Carriage	2.154	[0.0008, 0.2574, 0.0000]	[0.0829, 0.0077, 0.0756]
X Carriage	1.432	[−0.0418, −0.0077, −0.0032]	[0.0122, 0.0044, 0.0130]
B Rotation	1.046	[−0.1455, −0.0000, 0.0000]	[0.0062, 0.0036, 0.0037]
A Rotation (Including DU)	4.196	[−0.2445, −0.0039, 0.0000]	[0.0032, 0.0511, 0.0513]

**Table 5:** Joint stiffness and damping coefficient values.

Joint	Stiffness [N/m]	Damping Coefficient [N/(m/s)]
Suction cups (Planar joint)—compliance along X and Y	2.25 × 10^5^	15
Suction cups (planar joint)—rotational rigidity	2.5 × 10^6^	15
Suction cups (Prismatic joint)—stiffness in Z direction	3.37 × 10^6^	15
Carriage X-axis—under clamped boundary condition	4.51 × 10^5^	15
Carriage Y-axis—under clamped boundary condition	1.73 × 10^5^	15
A & B Axis	1.74× 10^5^	15

**Table 6:** Comparison of simulated and experimental modal frequencies and amplitudes, including RMSE and normalized errors.

Axis	Mode	Freq RMSE (Hz)	Norm. Freq Error (%)	Amp RMSE	Norm. Amp Error (%)
X	1st	2.7	8%	0.0372	623%
	2nd	2.6	4.2%	0.0312	135%
Y	1st	1.8	8%	0.0338	255%
	2nd	1.3	2.5%	0.0227	73.7%
Z	1st	4.8	3.6%	0.0601	74.4%

**Table 7:** CPS UI features and their corresponding ROS developments. Service servers (srv), clients (clnt), publishers (pub), and subscribers (sub) are listed from both the GUI perspective and the background calculations.

Feature	Topics	Description
Workpiece Model Upload	Selecting the workpiece STEP/STL file in the workpiece selection panel (clnt) /circle_explorer_service (srv)	The user is prompted to select the workpiece CAD file, upon which the path of the chosen workpiece is sent to/circle_explorer_service. This service finds the holes’ coordinates in the workpiece geometry using Open CASCADE.
Interactive Selection of the drilling holes	Interactive markers on the workpiece’s holes	Upon Open CASCADE finding the hole coordinates, cylindrical-shaped interactive markers are automatically placed. During task definition, the user may right-click on the markers to select the hole coordinates./circle_edge_markers is the associated node.
Interactive workpiece configuring	Interactive markers (arrows and rings) surrounding the workpiece/Draggable workpiece (srv, clnt)	Besides manual configuration, the operator is able to drag the workpiece within the visualization panel, i.e., the arrows and rings encircling the workpiece can be used to manipulate position (X, Y, Z) and orientation. The corresponding topic is/basic_controls.
Task selection	Combo box in Task Definition widget	If multiple tasks are defined, the operator selects the desired task to be executed from the combo box placed in the Task Definition widget, and it is sent to the ROS parameter server.
Command the carriage and the frame to go to the home position	Calibration QPushButton (clnt)	The end-user sets the homing process configuration in the corresponding dialog, after which a request will be made to the/homing server. Actuators bring the carriage and frame to the home position.
Execute and stop the drilling task	Execute ( 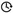 ) QPushButton (clnt)/drilling_service_server (srv)	After selecting the task, the operator may execute it, or in case of an emergency, the task can be stopped using the dedicated buttons. The designated server to execute the task is/drilling_service_server.
Joint synchronization of DT	Encoders of revolute/linear actuators (pub) /joint_synchronizer (sub)	Absolute and incremental encoders’ values of revolute and linear actuators are received while the ACME is moving. They are published to/joint_states_from_encoder, and the/joint_synchronizer node subscribes to it to update the DT’s joint values within the rendering panel in real-time.
Clamping force monitor	Clamping Force (sub) /drilling_service_server (pub)	/drilling_service_server reads the clamping force value from the loadcell and publishes it to the/loadcell_data. It also subscribes to the same topic to know when to stop clamping. /mainWindow also subscribes to/loadcell_data to display the clamping force in the GUI

**Table 8:** Summary of statistical parameters and process performance index (Ppk) for key quality metrics of the drilled holes.

Parameter	Positioning Error	Normality Error	Hole Diameter	Cylindricity
Mean (μ) [mm]	0.1901 mm	0.2891 °	5.0444 mm	0.02325 mm
Std. Dev (σ) [mm]	0.0930 mm	0.1381 °	0.012153 mm	0.00485 mm
LSL/USL	0.00/0.4 mm	−1.00/1.00 °	4.950/5.05 mm	0.00/0.05 mm
Ppk	0.681	1.716	0.154	1.599

**Table 9:** Performance of ACME based on the goal characteristics.

Characteristics	Achieved Performance	Established Thresholds in Design Phase	Ideal Thresholds in Design Phase	Pass
Weight (M)	18	20 kg	M≤15 kg	Pass
Setup Time ($T_{s}$)	3 min	3 min	$T_{a}\leq 2\;min$	Pass
Positioning time of the drilling head ($T_{p}$)	≤10 s from one side of workspace to the other	15 s	$T_{p}\leq 10\;s$	Pass
The Longer Dimension of the Workspace (LW)	~302 mm	300 mm	LW≥300 mm	Pass
Minimum clamping force ($F_{c}$)	Up to 1000 N	700 N	$F_{c}\geq 1000\;N$	Pass
Positioning Error (P-E)	Max = 0.34 mm	P-E:0.4 mm	P-E≤0.2 mm	Marginal
Normal Error (N-E)	Max = 0.49°	N-E: 1°	N-E ≤0.5°	Pass
Cost (C) [CAD]	~$20,000	$25,000	C≤$25,000	Pass

## Data Availability

The data supporting the findings of this study, including drilling results and sensor readings, are not publicly available at this time due to internal data management and confidentiality policies of the National Research Council Canada (NRC). However, these data can be made available upon reasonable request and subject to approval by NRC management.

## Supplementary Materials

The following supporting information can be downloaded at: https://www.mdpi.com/article/10.3390/s25247504/s1, Video S1: Demonstration of CPS functionalities and performance of ACME on double curvature fuselage surface.

## Abbreviations

The following abbreviations are used in this manuscript:

ACME	Advanced Collaborative Multifunctional End-Effector
Al	Aluminum
CAD	Computer-Aided Design
CAD$	Canadian Dollars
CFRP	Carbon Fiber-Reinforced Polymer
CMM	Coordinate Measuring Machine
Cobot	Collaborative Robot
CoM	Center of Mass
CPS	Cyber–Physical System
DH	Denavit–Hartenberg
DoF	Degree of Freedom
DOF	Degree(s) of Freedom
DT	Digital Twin
DU	Drilling Unit
EoAT	End-of-Arm Tool
FPK	Forward Pose Kinematics
FRF	Frequency Response Function
GUI	Graphical User Interface
HMI	Human–Machine Interface
HP	Horsepower
I/O	Input/Output
IPK	Inverse Pose Kinematics
NRC	National Research Council (Canada)
ODE	Ordinary Differential Equation
PWM	Pulse Width Modulation
ROS	Robot Operating System
RPM	Revolutions Per Minute
SRDF	Semantic Robot Description Format
STEP	Standard for the Exchange of Product Model Data
STL	Stereolithography File Format
TCP	Tool Center Point
Ti	Titanium
UR	Universal Robots (as robot manufacturer, e.g., UR10e)
URDF	Unified Robot Description Format
YAML	YAML Ain’t Markup Language
